# Identification of southern corn rust resistance QTNs in Chinese summer maize germplasm via multi-locus GWAS and post-GWAS analysis

**DOI:** 10.3389/fpls.2023.1221395

**Published:** 2023-09-21

**Authors:** Guoping Shu, Aifang Wang, Xingchuan Wang, Junqiang Ding, Ruijie Chen, Fei Gao, Aifen Wang, Ting Li, Yibo Wang

**Affiliations:** ^1^ Center of Biotechnology, Beijing Lantron Seed, LongPing High-tech Corp., Zhengzhou, Henan, China; ^2^ Henan LongPing-Lantron AgriScience & Technology Co., LTD, Zhengzhou, LongPing High-tech Corp., Zhengzhou, Henan, China; ^3^ College of Agronomy, Henan Agricultural University, Zhengzhou, Henan, China

**Keywords:** maize, multi-locus GWAS, QTNs, post-gwas, case-control, disease resistance gene, southern corn rust (SCR), *Puccinia polysora*

## Abstract

Southern corn rust (SCR) caused by *Puccinia polysora* Underw is a major disease leading to severe yield losses in China Summer Corn Belt. Using six multi-locus GWAS methods, we identified a set of SCR resistance QTNs from a diversity panel of 140 inbred lines collected from China Summer Corn Belt. Thirteen QTNs on chromosomes 1, 2, 4, 5, 6, and 8 were grouped into three types of allele effects and their associations with SCR phenotypes were verified by post-GWAS case-control sampling, allele/haplotype effect analysis. Relative resistance (RR_R_) and relative susceptibility (RRs) catering to its inbred carrier were estimated from single QTN and QTN-QTN combos and epistatitic effects were estimated for QTN-QTN combos. By transcriptomic annotation, a set of candidate genes were predicted to be involved in transcriptional regulation (*S5_145, Zm00001d01613*, transcription factor GTE4), phosphorylation (*S8_123, Zm00001d010672*, *Pgk2-* phosphoglycerate kinase 2), and temperature stress response (*S6_164a/S6_164b, Zm00001d038806, hsp101*, and *S5_211, Zm00001d017978, cellulase25*). The breeding implications of the above findings were discussed.

## Introduction

Southern corn rust (SCR), caused by the fungus *P. polysora*Underw., is a major disease that leads to significant grain yield loss in maize (*Zea mays* L.). SCR is common in warm-temperate and tropical regions of the world, and is a severe maize disease in regions such as China’s Summer Corn Belt (SCB, also known as the Huanghuaihai Summer Corn Region), the SCB of the United States, and Africa (Storey and Ryland, 1954; Dong et al., 2010; Brewbaker et al., 2011; Wang et al., 2014; Zhao et al., 2018; Duan et al., 2020; Wang et al., 2020b; Sun et al., 2021b). In China, SCR was first identified in Sanya and Ledong, Hainan Province, in early 1972 (Duan and He, 1984) and has gradually spread to high-latitude areas because of global climate change (Wang et al., 2020b). A major outbreak of SCR in China’s SCB occurs every 2–3 years and has resulted in grain yield losses of up to 50% (Zhou et al., 2008; Wang et al., 2014; Lv et al., 2020). A lack of resistance genes and a narrow germplasm basis are the main reasons for SCR epidemics (Brewbaker et al., 2011; Chen et al., 2018; Chen et al., 2019; Deng et al., 2022). Recent surveys of Chinese maize germplasm have shown that only a small number of varieties (less than 15% of commercial maize varieties and newly released varieties) have high resistance to SCR, and the majority of commercial maize hybrids are susceptible (Wang, 2005; Wang et al., 2014; Sun et al., 2021b), including some major commercial varieties cultivated in China’s SCB, such as Zhengdan 958 and Xianyu 335. It is not only desirable but also urgent to identify more resistance genes from diverse germplasm resources.

Genetic improvement is the most important strategy for reducing maize yield losses caused by SCR in China (Ali and Yan, 2012; Wang et al., 2014). A number of genes and QTLs with major phenotypic effects have been identified using linkage-based genetic mapping populations, including *Rpp1*, *Rpp9*, *RppP25*, *RppM*, *RppK*, and *RppCML496* (Zhou et al., 2017; Deng et al., 2019; Deng et al., 2020; Lv et al., 2020; Wang et al., 2020a); these loci are all located at the short arm of chromosome 10, are believed to be race-specific, and the associated resistance is easily lost with a change in the *P. polysora*Underw. fungus (Xiao et al., 2017; Sun et al., 2021b). Mining of genes with a broad resistance spectrum has not been very successful using linkage-based genetic mapping populations, either with temporary segregation populations (Mu et al., 2022; Li et al., 2023b) or with permanent segregation populations such as RILs (Wanlayaporn et al., 2013). The genome-wide association study (GWAS) approach is widely used in identifying small-effect loci of a complex trait in human genetics (Uffelmann et al., 2021) and has also been used for other maize diseases (Shikha et al., 2021; Sun et al., 2021b). However, this approach has not been widely used in genetic mapping of SCR resistance (Zhou et al., 2017; De Souza Camacho et al., 2019; Li et al., 2023b). GWAS is also known to generate a large proportion of false positive signals.

In this report, we identify a set of SCR-resistance QTNs based on multi-locus GWAS from a diversity panel of 140 inbred maize lines that include most of the parental lines of major commercial hybrids in the China Summer Corn Belt. The SCR-resistance QTNs identified were further verified by two post-GWAS analyses: case–control sampling and allele/haplotype effect analysis. Resistant and susceptible alleles were then evaluated for relative resistance and relative susceptibility, with the goal of employing them in molecular breeding of SCR-resistant varieties of maize.

## Materials and methods

### Genetic materials and SCR phenotyping

In a previous study (Shu et al., 2021), 490 inbred lines collected from maize breeders at China Summer Corn Belt were genotyped via genotyping-by-sequencing (GBS); among these, 140 inbred lines that can grow and mature normally in both the summer corn region and Sanya winter nursery in Hainan Province were scored for resistance to southern corn rust (SCR). All 140 of these inbred maize lines were planted in a complete randomized design at Nanbin Station (N18°21′7′′, E109°10′20′′), Sanya, Hainan province, China, in November 2013, in two-row plots of 5 m length and spaced 0.60 m apart, with 25 plants per row. Standard crop management practices were followed throughout the growing season to ensure normal growth without weed or insect damage. Natural infection with airborne uredospores of *P. polysora* was employed to induce the disease phenotype. To provide an abundant supply of the SCR pathogen, highly SCR-susceptible inbred lines Ye478 and Lx9801 were planted in a rectangular lattice across the field surrounding the SCR-phenotyping plots; this controlled field test plot design has been confirmed as an effective way to ensure efficient spread of the pathogen to neighboring plants in fields (Jines et al., 2007; Zhao et al., 2013) and has been adopted as the standard method of SCR phenotyping in maize breeding and genetic research in China (Zhao et al., 2013; Deng et al., 2020; Lv et al., 2020; Wang et al., 2020b). Disease scores were recorded at 30 days post-pollination. The degree of infection on each plant was visually scored on a scale of 1 to 9 using the Stakman infection type scale with 1 = HR (highly resistant, almost free of rust pustules), 3 = R (resistant, infection covering 6% to 10% of leaf area), 5 = MR (moderately resistant, infection covering 11% to 30% of leaf area), 7 = S (susceptible, infection covering 31% to 70% of leaf area), and 9 = HS (highly susceptible, nearly completely covered with rust pustules) (Wang et al., 2014; Deng et al., 2019; Deng et al., 2020; Li et al., 2021). The scores for each plot, based on the average of all plants in the plot, were used as the phenotype scores for each inbred line.

### DNA sequencing and genotyping

A leaf sample from each inbred line was used for DNA extraction via a CTAB procedure. The protocol reported by Elshire et al. (2011) was followed for DNA sequencing. Briefly, genomic DNA was digested with the restriction enzyme ApeK1, and genotyping-by-sequencing (GBS) libraries were then constructed in 96-plex and sequenced on an Illumina HiSeq 2000. SNP calling was performed using the TASSEL-GBS pipeline (Glaubitz et al., 2014), with B73 RefGen V2.0 as the reference genome. Initially, 87,6297 SNPs were filtered with minor allele frequency (MAF) > 5%, missing rate< 20%, and heterozygosity rate< 25% (Shu et al., 2021); data on 68,768 high-quality SNP loci were retained for entry into all analyses conducted in this study.

### Population structure, linkage disequilibrium, and multi-locus GWAS

ADMIXTURE 1.3.0 (http://dalexander.github.io/admixture/download.html) was used to determine the population structure among all 140 inbred maize lines using 68,768 SNPs (Alexander and Lange, 2011), and the “Expectation Maximization” clustering algorithm was run with cluster numbers ranging from K = 1 to K = 12 in order to determine the optimum number of clusters K producing the smallest cross-validation error. Linkage disequilibrium (LD) analyses were carried out using TASSEL 5.2.25 (https://www.maizegenetics.net/tassel) (Bradbury et al., 2007; Glaubitz et al., 2014). The following six multi-locus GWAS methods from the R software package mrMLM.GUI V4.0.2 (Zhang et al., 2019; Zhang et al., 2020) were used to detect significant QTNs for SCR resistance: (1) mrMLM (https://cran.r-project.org/web/packages/mrMLM/index.html, Wang et al., 2016); (2) FASTmrEMMA (Wen et al., 2018); (3) ISIS EM BLASSO (Tamba et al., 2017); (4) pLARmEB (Zhang et al., 2017a); (5) pKWmEB (Ren et al., 2018); and (6) FASTmrMLM (Tamba & Zhang, 2018, bioRxiv). Q-matrices generated using ADMIXTURE 1.3.0 and K-matrices generated using mrMLM.GUI V4.0.2 were applied to correct the population structure and polygenic backgrounds. An intermediate result file and a final result file were generated. Manhattan plots were generated based on the intermediate and final results files using mrMLM.GUI V4.0.2.

### Case control sampling

Case–control sampling is a common design in human genetic mapping, in which cases and controls are defined based on the presence or absence of a certain phenotype, respectively (Uffelmann et al., 2021). In this study, case–control sampling was employed to verify the phenotype–SNP association detected in GWAS. Briefly, 10 inbred lines with the highest SCR disease phenotype scores (all with phenotype score 9) were sampled as cases, and 10 inbred lines with the lowest SCR disease phenotype scores (all with phenotype score 1) were sampled as controls; the SNP allele genotype and the mean phenotypic value for the corresponding allele for each QTN identified in multi-locus GWAS were compared between the case sample and the control sample for consistency. The phenotypes are visualized on a color scale in (with phenotype values from 1–9 expressed on a color scale from blue to red).

### Allele/haplotype effect analysis, QTN/combo performance estimates, and epistasis

#### Allele/haplotype effect estimates

The phenotype effect of an allele of a single QTN or a haplotype of combos generated from two QTNs, called the allele/haplotype effect, was estimated using the average SCR score of their carrier inbred lines: allele/haplotype effect = average SCR score of resistance allele/haplotype carriers (SCR_R_) – average SCR score of susceptible allele/haplotype carriers (SCR_S_).

#### Allele/haplotype effect type assignment

The allele/haplotype effect type of each QTN was categorized as HR, R, MR, S, or HS. Here, these labels corresponded to allele/haplotype phenotypic values of 1.0–1.9, 2.0–3.9, 4.0–5.9, 6.0–7.9, and 8.0–9.0, respectively.

#### QTN/combo performance estimate

For each QTN, the relative risk statistic (RR) was calculated as a measure of the performance (in terms of relative resistance, RR_R_) of its resistant allele/haplotype over its susceptible allele/haplotype, and the relative susceptibility (RR_S_) of its susceptible allele/haplotype over its resistant allele/haplotype; these statistics were calculated using the following equations:

(1) Relative resistance of resistant allele/haplotype over susceptible allele/haplotype:


(1) $${\text{RR}}_{\text{R}}=\left({\text{N}}_{11}/\left({\text{N}}_{11}+{\text{N}}_{12}\right)\right)/\left({\text{N}}_{21}/\left({\text{N}}_{21}+{\text{N}}_{22}\right)\right)$$

(2) Relative susceptibility of susceptible allele/haplotype over resistant allele/haplotype:


(2) $${\text{RR}}_{\text{S}}=\left({\text{N}}_{22}/\left({\text{N}}_{21}+{\text{N}}_{22}\right)\right)/\left({\text{N}}_{12}/\left({\text{N}}_{11}+{\text{N}}_{12}\right)\right)$$

In this case, the total inbred lines (N) from the GWAS analysis were divided into resistance allele carriers (N_1_) and susceptible allele carriers (N_2_); among N_1_ carriers, N_11_ and N_12_ represent the number of resistant allele carriers that have the resistant and susceptible phenotypes, respectively; similarly, among N_2_ carriers, N_21_ and N_22_ represent the number of susceptible allele carriers that have the resistant and susceptible phenotypes, respectively.

#### Epistasis

Epistasis (also known as the epistatic effect or modifying effect) was estimated for each QTN pair or combo using the –epistasis function of PLINK v1.07 (Purcell et al., 2007) by executing the following commands at the command line: > plink –file “input filename” –pheno phenoq.txt –epistasis –epi1 1 –noweb–out “output filename”. The use of –epi1 1 enabled the generation and output of epistatic effect estimates for all QTN pairs, shown in **Table S1**.

### Identification of candidate genes and tissue-specific and stress-induced transcriptomic analysis

FASTA sequences containing significant QTNs were re-aligned to the B73 v4 reference genome in order to obtain a more accurate physical localization for improved gene annotations (https://www.maizegdb.org/gbrowse). For each statistically significant QTN identified via multi-locus GWAS, the annotated genes within ±300kb of the chromosome region (based on B73 Reference V4) were considered as candidate genes, as described in Ahmed et al. (2022). The location of a QTN in a gene and its effect were analyzed using the ANNOVAR software package (Wang et al., 2010). The polymorphic SNPs surrounding key significant QTNs, their SCR phenotype associations, and their association with gene structures were explored using scatter plots and gene structure diagrams. The expression profiles for each candidate gene in different organs and tissues at different developmental stages and under exposure to different biotic and abiotic stresses were extracted from the transcriptomic databases at MaizeGDB (https://www.maizegdb.org/). The relationship between expression of each candidate gene and resistance to southern corn rust was analyzed.

## Results

### SNP genotype data characterization

**Figure 1:**
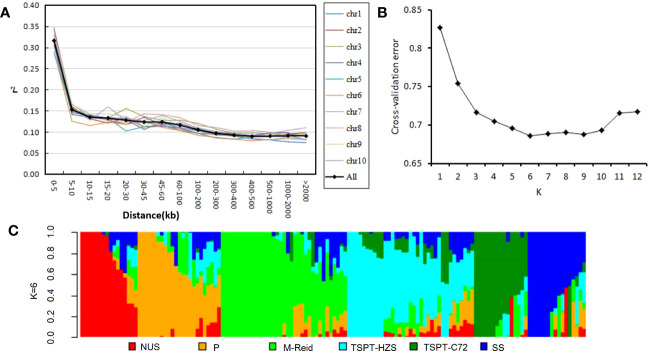
Linkage disequilibrium decay and population structure in the diversity panel of 140 inbred lines. **(A)** Linkage disequilibrium decay across all 10 maize chromosomes. **(B)** Cross-validation error values for K = 1–12 in population structure analysis. **(C)** Population structure of the 140 inbred lines at K = 6. NUS, new US germplasm; P, P78599; M-Reid: modified-Reid; TSPT_HZS, TangSiPingTou_Huangzaosi; TSPT_C72, TangSiPingTou_Chang7-2; SS, stiff stalk.

Using the control procedure of the Tassel pipline (see Materials and Methods section), the quality of the genotype data of the set of 140 inbred lines was improved by filtering out SNPs with minor allele frequency (MAF)< 5% and SNPs with more than 20% of the genotyping data missing (**Figure S1**). The filtered genotype data of 68,769 SNP loci were evaluated for LD decay on all 10 maize chromosomes (**Figure 1A**); the mean LD decay distance across all chromosomes for this diversity panel was r^2^ = 0.1, or approximately 200~300 kb (**Figure 1A**). The 140 inbred lines were evaluated for population structure using the SNP data (see Material and Methods section). ADMIXTURE 1.3.0 was used to detect population structure; the cross-validation error was smallest at K=6 (**Figure 1B**), indicating there were 6 subgroups. This population structure is visualized in **Figure 1C**.

### Multi-locus GWAS and QTN identification

Genome-wide association study (GWAS) was conducted using SCR phenotype data, SNP genotype data from the diversity panel of 140 inbred lines, and the population structure information through use of a multi-locus GWAS R package that offers six different statistical and computational models (see Material and Methods section). All statistically significant QTNs, including 21 QTNs identified by GWAS (defined as a LOD score greater than 3.00), are reported in **Supplementary Table S1**; among these, 13 QTN loci associated with putative candidate genes are listed in **Table 1**. Pairwise LD values among 10 QTNs are shown in **Supplementary Figure S2**. Among the 21 QTNs identified by GWAS (**Supplementary Table S1**), eight QTNs were located on exons, six in intergenic regions, three in the upstream regions of genes, two on introns, one in the UTR3 region, and one in the UTR5 region. Among the eight QTNs located on exons, three variants located on *Zm00001d032244*, *Zm00001d033259*, and *Zm00001d052781* were identified as nonsynonymous SNVs causing an amino acid change, whereas 5 variants were identified as synonymous SNVs not causing an amino acid change (**Table S1**).

**Table 1:** QTNs for southern corn rust (SCR) resistance identified by multi-locus GWAS models and annotations.

QTN ID	Chr #	Position (B73 RefGen V4.0)	Bin	GWAS model	QTN effect	LOD score	r^2^ (%)	Candidate genes	Location of SNPs in genes	Gene symbol	Description
*S1_218*	1	218619398	1.07	4, 6	1.16	4.58	8.10	*Zm00001d032240,Zm00001d032244*	Exon/non-synonymous	*myb146*	MYB-transcription factor 146, probable inactive receptor kinase
*S1_299b*	1	299623487	1.11	3	-1.20	8.58	18.13	*Zm00001d034678*	UTR5	*nbcs4*	nucleobase-cation symporter 4
*S2_12*	2	12916090	2.01	2, 6	-0.81	5.07	4.06	*Zm00001d002447*	Intergenic	*rlk12*	receptor-like protein kinase 12
*S2_220*	2	220613149	2.08	6	-0.73	3.98	5.13	*-*	Intergenic	*-*	–
*S4_170*	4	170324384	4.06	2, 3, 6	-0.53	3.99	3.94	*Zm00001d051812*	Intronic	*hk6*	histidine kinase 6
*S4_200*	4	200738088	4.08	3	0.55	4.82	4.15	*Zm00001d052781*	Exon/non-synonymous	*cct23*	CO CO-LIKE TIMING OF CAB1 protein domain 23
*S5_145*	5	145816043	5.04	4	1.36	3.47	11.16	*Zm00001d016131*	Synonymous	*GTE4*	Transcription factor GTE4
*S5_210*	5	210212211	5.06	2, 6	1.09	5.92	9.28	*Zm00001d017927*	Upstream	*Fcf2*	Fcf2 pre-rRNA processing protein
*S5_211*	5	211183684	5.06	1, 3, 4	1.26	6.81	11.69	*Zm00001d017978*	Exon/synonymous	*cel25*	cellulase 25
*S6_164a*	6	164808768	6.07	4	0.63	3.47	4.09	*Zm00001d038791,Zm00001d038806*	Exon/synonymous	*rlk10, hsp101*	receptor-like protein kinase 10, heat-shock protein 101
*S6_164b*	6	164811804	6.07	2	1.63	4.47	5.09	*Zm00001d038791,Zm00001d038806*	UTR3	*rlk10, hsp101*	receptor-like protein kinase 10, heat-shock protein 101
*S6_165*	6	165682422	6.07	6	0.60	5.22	4.62	*Zm00001d038843*	Intergenic	*wrky82*	WRKY-transcription factor 82
*S8_123*	8	123503579	8.04	2, 6	0.70	4.13	6.71	*Zm00001d010672, Zm00001d010673*	Intergenic	*pgk2*	pgk2 - phosphoglycerate kinase 2 (also known as metacaspase type II)

GWAS Models 1, 2, 3, 4, 5, and 6 refer to multi-locus models FASTmrEMMA, FASTmrMLM, ISIS EM-BSSO, mrMLM, pKWmEB, and pLARmEB, respectively.

R^2^ represents phenotypic variation explained.

**Figure 2:**
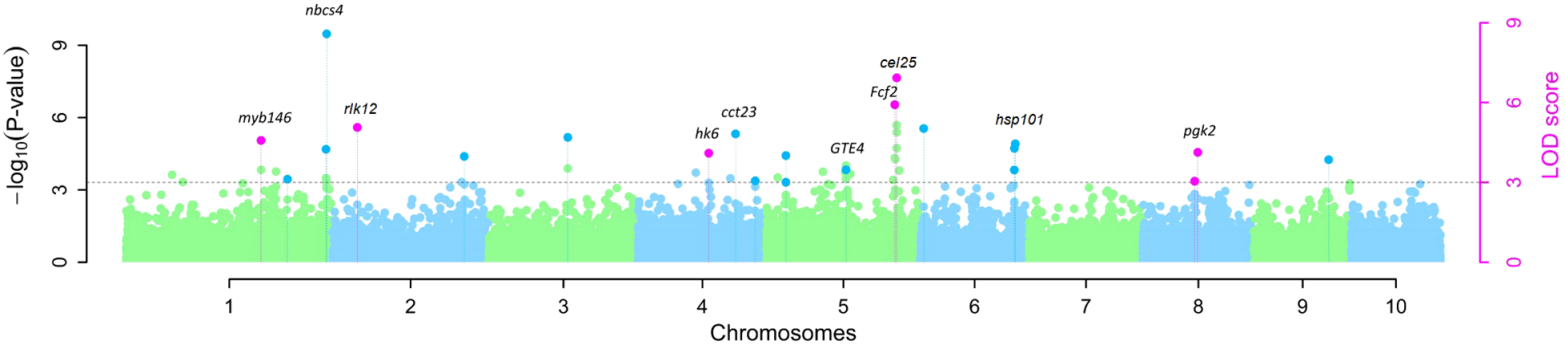
Manhattan plot of QTNs for resistance to southern corn rust (SCR) detected by GWAS in 140 inbred lines of maize. QTNs identified by multiple models are indicated by pink dots with vertical lines; QTNs identified by a single model are indicated by light green or blue dots with vertical lines.

The map positions of the 13 QTNs reported in **Table 1** were also labeled on a Manhattan plot (**Figure 2**) produced using the R software package mrMLM.GUI V4.0.2 (see Materials and Methods section). As shown in **Table 1**; **Supplementary Table S1**; **Figure 2**, the 13 significant QTNs were located on Chromosomes 1, 2, 4, 5, 6, and 8; some chromosomal regions were found to contain multiple tightly linked QTNs, such as bin1.11 (QTNs *S1_299a* and *S1_299b* (*nbcs4*)), bin5.06 (QTNs *S5_210* (*Fcf2*) and *S5_211*(*cel25*)), and bin6.07 (QTN *S6_164a* (*hsp101*) and *S6_164b* (*hsp101*)). Three QTNs that were found to explain more than 10% of the phenotypic variation (r^2^%) are shown in **Table 1**; these are *S1_299b* (*nbcs4*), *S5_145* (*GTE4*), and *S5_211* (*cel25*).

### Allele effect type assignment

As shown in **Table 2**, the two alleles from each QTN were associated with significantly different phenotypic effects on their carriers. Based on measures of the difference in allelic effect, the 13 QTNs fell into four different categories or allele effect types: (1) type A, or HR/S type: one allele is associated with high resistance (HR) and the other with susceptibility (S); (2) type B, or MR/S type: the two alleles are associated with moderate resistance (MR) and susceptibility (S), respectively; (3) type C, or MR/HS type: the two alleles are associated with moderate resistance (MR) and high susceptibility (HS), respectively; and (4) type D, or S/HS type: the two alleles are associated with susceptibility (S) and high susceptibility (HS), respectively. From **Table 2**, it can be seen that most of the highly resistant (HR) inbred lines, such as Qi319, X178, P138, CT01, and CT1251, carry not only HR alleles of type A QTNs (*S5_145*, *S5_210*), but also either MR alleles of type B and type C QTNs or S alleles of type D QTNs; in contrast, most of the highly susceptible (HS) inbred lines, such as Zong3, Zheng58, 478, and Xun926, carriers of the HS alleles of type D QTNs (**Tables 2**, **3**).

**Table 2:** Resistant and susceptible alleles of significant QTNs and their inbred carriers.

QTN type	QTN ID	Chr#	Position (B73 V4.0)	Effect type	Allele (R/S)	Allele effect (R/S)	N_1_/N_2_	Resistant allele carriers	Susceptible allele carriers
Type A	*S5_145*	5	145816043	HR/S	GG/AA	1.8/7.6	10/125	Qi319, X178, P138, CT1251, PT46, L19-SSS	CT01, Xun248, F06, Chang7-2, 581, Jing24, 9801H, TSPT, Huangzaosi, CT201-1S111, Xun926, Huangyesi, Ji853
Type A	*S5_210*	5	210212211	HR/S	GG/CC	1.8/7.6	10/120	Qi319, X178, P138, CT01, CT1251, L19-SSS, F06	PT46, Xun248, Chang7-2, 581, Jing24, 9801H, TSPT, Huangzaosi, CT201-1S111, Xun926, Huangyesi, Ji853
Type B	*S1_218*	1	218619398	MR/S	AA/GG	4.3/7.3	8/125	CT1251, CT01, Xun248, DH351, CT02	Zong3, Ji868, Zheng58, WK858, Yuanwu02
Type B	*S6_165*	6	165682422	MR/S	AA/GG	5.1/7.8	27/101	Qi319, PT46, CT019-21, L19-SSS, F06	CT01, CT1251, Xun248, Chang7-2, 581, Jing24, 9801H, TSPT, Huangzaosi, CT201-1S111, Xun926, Huangyesi, Ji853
Type C	*S5_211*	5	211183684	MR/HS	GG/TT	5.3/8.0	42/88	Qi319, X178, P138, CT01, CT1251, CT019-21, L19-SSS	PT46, Xun248, Chang7-2, 581, Jing24, 9801H, TSPT, Huangzaosi, CT201-1S111, Xun926, Huangyesi, Ji853
Type C	*S8_123*	8	123503579	MR/HS	TT/GG	4.4/8.0	16/95	Qi319, PT46, L19-SSS	F06, 581, Jing24, 9801H, TSPT, Huangzaosi, CT201-1S111, Xun926, Huangyesi, Ji853
Type D	*S1_299b*	1	299623487	S/HS	GG/TT	6.8/8.3	102/12	Qi319, X178, CT01, CT1251, PT46, CT019-21, L19-SSS, Xun248	NC20, 6WC, NH60, LN287, 09B, PHW52, SNNdN, Ye515, Ji868, H446, Ye8112, Tie7922
Type D	*S2_12*	2	12916090	S/HS	AA/GG	6.9/9.0	125/10	Qi319, X178, P138, CT01, CT1251, PT46, CT019-21, L19-SSS, Xun248, F06	L811, DHuang212, Zong3, PC7-23111S111, 207, Yuanwu02, Huangyesi, K7M129, LDA801, Dan360
Type D	*S2_220*	2	220613149	S/HS	GG/AA	7.0/9.0	114/6	X178, P138, CT01, CT1251, PT46, CT019-21, Xun248, F06	Jing7H, Jing24, 478, WNxNN, S7M114, K7M129
Type D	*S4_170*	4	170324384	S/HS	TT/AA	6.9/8.0	93/16	Qi319, X178, CT01, CT1251, PT46, CT019-21, Xun248, F06	w6k8y8F, Ji53, HuangC, Zh7922, Ye488, L189, Xun9058, Jing724, Hu803, E28, Lvjiuk, 478, 54-0, Zheng22, S7M114, Dan360
Type D	*S4_200*	4	200738088	S/HS	GG/CC	6.6/8.1	89/44	Qi319, X178, CT01, CT1251, PT46, CT019-21, Xun248, F06	Jing724, Ji853, Dan340, Zong3, WK858
Type D	*S6_164a*	6	164808768	S/HS	GG/AA	6.6/8.5	83/29	Qi319, X178, P138, CT01, CT1251, PT46, CT019-21, Xun248, F06, Jing24, TSPT, Huangzaosi, Huangyesi, Ji853	581, 9801H, CT201-1S111, Xun926, Zheng58, Jing92, Jing724
Type D	*S6_164b*	6	164811804	S/HS	GG/AA	6.8/8.2	103/25	Qi319, X178, P138, CT01, CT1251, PT46, CT019-21, Xun248, F06, Jing24, TSPT, Huangzaosi, Huangyesi, Ji853	581, 9801H, CT201-1S111, Xun926, Zheng58, Jing92, Jing724

N_1_, number of resistant allele carriers, N_2_, number of susceptible allele carriers.

**Table 3:** QTN type, allele/haplotype effect, and relative resistance and relative susceptibility performance.

QTN type	QTN ID	Allele_R/S	SCR_R_	SCR_S_	Allele/haplotype effect	N_11_	N_12_	N_21_	N_22_	RR_R_	RR_S_
Type A	*S5_145*	GG/AA	1.8	7.6	-5.8	10	0.5	20	105	5.95	17.64
Type A	*S5_210*	GG/CC	1.8	7.6	-5.8	10	0.5	21	99	5.44	17.33
Type B	*S6_165*	AA/GG	5.1	7.8	-2.7	15	12	14	87	4.01	1.94
Type B	*S1_218*	AA/GG	4.3	7.3	-3.0	5	3	27	98	2.89	2.09
Type C	*S8_123*	TT/GG	4.4	8.0	-3.6	13	3	8	87	9.65	4.88
Type C	*S5_211*	GG/TT	5.3	8.0	-2.7	23	19	9	79	5.35	1.98
Type D	*S1_299b*	GG/TT	6.8	8.3	-1.5	30	72	0.5	12	7.35	1.36
Type D	*S2_12*	AA/GG	6.9	9.0	-2.1	34	91	0.5	10	5.71	1.31
Type D	*S2_220*	GG/AA	7.0	9.0	-2.0	30	84	0.5	6	3.42	1.25
Type D	*S4_170*	TT/AA	6.9	8.0	-1.1	26	67	1	15	4.47	1.3
Type D	*S4_200*	GG/CC	6.6	8.1	-1.5	29	60	4	40	3.58	1.35
Type D	*S6_164a*	GG/AA	6.6	8.5	-1.9	27	56	1	28	9.43	1.43
Type D	*S6_164b*	GG/AA	6.8	8.2	-1.4	29	74	2	23	3.52	1.28
Combo 1	*S6_164a.S6_164b*	GGGG/AAAA	6.6	8.4	-1.8	25	53	1	18	6.09	1.39
Combo 2	*S6_165.S8_123*	AATT/GGGG	2.4	8.4	-6.0	7	0.5	2	70	33.6	14.58
Combo 3	*S5_145.S5_210*	GGGG/AACC	1.3	7.8	-6.5	6	0.5	15	99	7.02	11.29
Combo 4	*S5_211.S8_123*	GGTT/TTGG	4.5	8.4	-3.9	9	2	1	68	56.45	5.42
Combo 5	*S6_165.S6_164a*	AAGG/GGAA	4.8	8.4	-3.6	13	9	1	23	14.18	2.34
Combo 6	*S6_165.S6_164b*	AAGG/GGAA	4.7	8.2	-3.5	13	9	1	19	11.82	2.32
Combo 7	*S5_211.S6_165*	GGAA/TTGG	4.5	8.3	-3.8	8	5	3	69	14.77	2.49
Combo 8	*S2_12.S5_210*	AAGG/GGCC	1.8	9.0	-7.2	10	0.5	0.5	6	12.38	19.38

see Materials and Methods section for further details on calculations. N_11_, number of resistance allele carriers with resistance phenotype; N_12_, number of resistance allele carriers with disease phenotype; N_21_, number of susceptible allele carriers with resistance phenotype; N_22_, number of susceptible allele carriers with disease phenotype. RR_R_ and RR_S_ are the relative resistance of the resistant allele over the susceptible allele and the relative susceptibility of the susceptible allele over the resistant allele, respectively. If N=0, it was replaced with 0.5 for the purpose of calculations.

### Allelic and haplotype effect analysis

**Figure 3:**
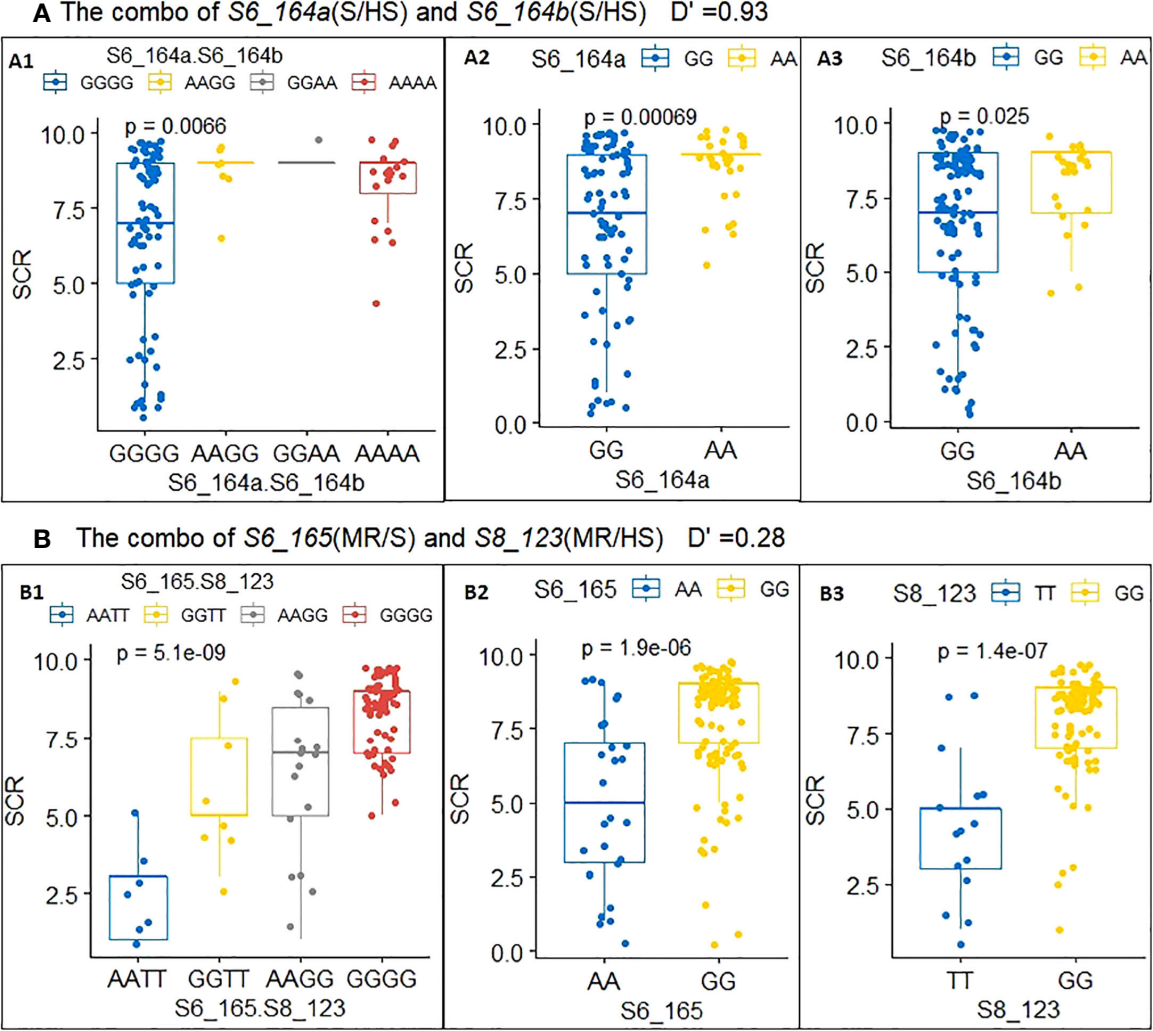
Allele and haplotype phenotypic effects of a single QTN and multi-QTN combos with low and high LD. **(A)** Combo 1: S6_164a.S6_164b(A1), S6_164a (A2) and S6_164b(A3). **(B)** Combo 2: S6_165.S8_123(B1), S6_165 (B2) and S8_123(B3).

The allele phenotypic effect of each single QTN (**Table 3**) and the joint phenotypic effects of haplotypes from two QTNs (known as a QTN combo) were estimated via allele/haplotype effect analysis (**Table 3**); these effects were also visualized using boxplots, two examples of which are shown in **Figure 3**. QTN *S6_164a* and *S6_164b* (**Table 3**, combo 1) were two tightly linked QTN loci with D′= 0.93 (**Supplementary Figure S1**), and physically were only 3036 bp apart on Chr6 (see B73 RefGen V4 position in **Table 2**); both were type D QTN loci (S/HS allele effect type), and both were found to have AA and GG alleles (**Figures 3**-A2, 3-A3). In order to evaluate their joint phenotype effects, the haplotype effects for all four observed haplotypes (GGGG, AAGG, GGAA, AAAA), in the form of the average SCR score of their carrier inbred lines, were plotted as shown in **Figure 3**-A1. The haplotypes GGGG and AAAA were found have an almost identical SCR phenotypic effect to that of the GG and AA alleles of a single QTN alone (**Figures 3**-A2, 3-A3); the haplotype AAGG and GGAA had very few counts, indicating that these are likely recombinant types, and the GGGG and AAAA are likely parental types. The joint phenotypic effects of combo 2 (*S6_165.S8_123*) are shown in **Table 3** and in **Figure 3**-B1, 3-B2, 3-B3. *S6_165* (MR/S) and *S8_123* (MR/HS) were found to be two almost completely independent QTNs (D′= 0.28, **Figure S1**), producing four observed haplotypes (AATT, GGTT, AAGG, GGGG); as shown in **Figure 3**-B1 and **Table 3**, the haplotype AATT carriers were found to have an SCR phenotypic effect of 2.4, and were more likely to be resistant (R) inbred lines than either the AA allele carriers of QTN *S6_165* (5.1, **Figure 3**-B2; **Table 3**) or the TT allele carrier of *S8_123* (4.4, **Figure 3**-B3; **Table 3**), both of which were found to have an SCR phenotypic effect of around 5.0. Similarly, the GGGG haplotype was found to be a better predictor of an HS phenotype in its carriers than the GG allele of *S6_165* or the GG allele of *S8_123* alone (**Figure 3**-B1, 3-B2, 3-B3, **Table 3**). As shown in **Table 3**, the maximum haplotype effect of combo 2 (*S6_165.S8_123*, AATT vs GGGG) was -6.0, much larger than the allele effect of QTN *S8_123* (AA vs GG, -3.6) or QTN *S6_165* (TT vs GG, -2.7) alone by absolute value. In comparing the two combos, it is clear that combo 2 has much better predictive value, because the two QTNs have low LD and they can freely shuffle to generate a more advantageous allele combination or haplotype than combo 1.

### Case–control sampling

**Figure 4:**
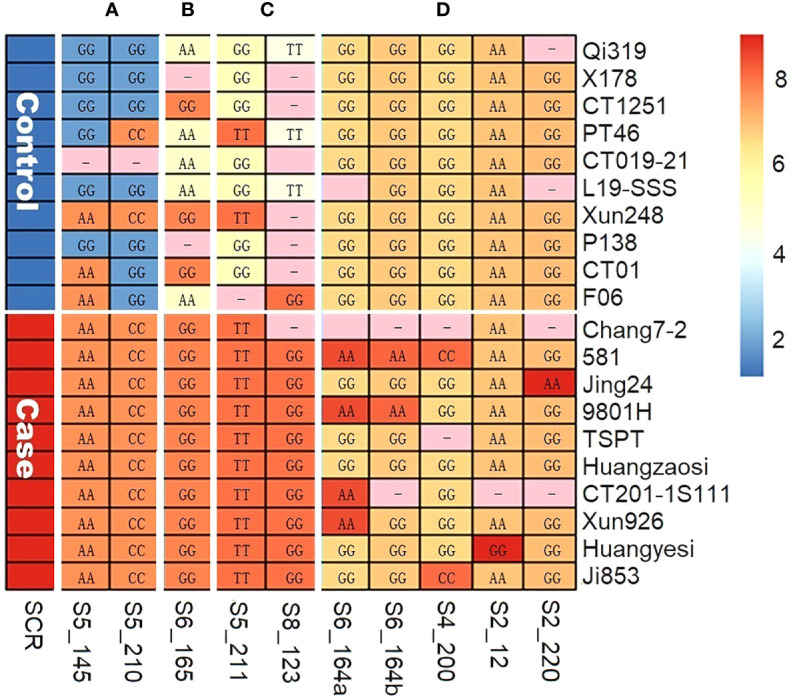
The allele genotypes (letters) and allele effect phenotypes (colors) of 10 QTNs, as observed in case–control sampling. **(A)** QTNs of type A. **(B)** QTN of type B. **(C)** QTNs of type C. **(D)** QTNs of type D.

To verify the significant association between the SCR phenotype and QTN genotype that we had observed in multi-locus GWAS, a case–control sampling study was conducted (see Materials and Methods section). Specifically, 10 case inbred lines and 10 control inbred lines were genotyped for 10 QTN loci; the results are summarized in **Figure 4**, where rows represent inbred carrier, columns represent QTNs, and the letters and the color of each cell *C_ij_* represent the SNP genotype and the allelic phenotypic value of QTN *j* of inbred carrier *i*, respectively. The allelic effect type (A, B, C, or D, from **Table 2**) for each QTN are indicated in the top margin of **Figure 4**. Based on **Figure 4**; **Table 2**, our findings were as follows: (1) at type A QTNs, all case inbred lines carried S alleles in type A and type B QTNs and had a susceptible allelic effect phenotype (shown by the orange color), whereas all control inbred lines carried HR alleles and had a highly resistant allelic effect phenotype (shown by the blue color) at type A QTNs, with the exception of Xun248, which was found to carry only S alleles of type A, and CT019-21, for which genotype data were missing; (2) all case inbred lines carried HS alleles and had highly susceptible allelic effect phenotype (shown by the red color) at two type C QTNs and at one or more type D QTNs, while none of the control inbred lines carried HS alleles of type D QTNs. As shown in **Figure 4**, the associations between the allelic genotypes carried by the case and control inbred lines and the phenotypes observed were highly consistent across different QTNs.

### Resistance performance and disease risk analysis

For each of the 13 QTNs and the 8 combos of two QTNs, the allelic (haplotype) effect, expressed as the difference between its resistant alleles (or haplotypes) and its susceptible alleles (or haplotypes) in terms of average SCR phenotype score is reported in **Table 3**; a negative value indicates that the resistant alleles (or haplotypes) can reduce the SCR score of their carriers, thereby enhancing the carrier’s disease resistance performance. Among single QTNs, the two type A QTNs had the largest allele effect by absolute value, with a value of -5.8. Type D QTNs had the smallest allelic effect by absolute value (ranging from -2.1 to -1.1; **Table 3**). Among combos of two QTNs, the combo of two type A QTNs (combo 3) had an effect of -6.5, larger than any single type A QTN, while the combo of one type D QTN and one type A QTN (combo 8, *S2_12.S5.210*) had the largest allele effect, -7.2. For each QTN, the relative performance of its resistant allele over its susceptible allele was measured in the form of RR_R_, and the relative performance of its susceptible allele over its resistant allele was measured in the form of RR_S._ As shown in **Table 3**, for any QTN, its resistant allele significantly enhanced disease resistance performance over its susceptible allele, with RR_R_ values ranging from 2.89- to 9.65-fold, and its susceptible alleles significantly increased disease risk over its resistant allele, with RR_S_ values ranging from 1.25- to 17.64-fold. For combos of two QTNs, RR_R_ values ranged from 6.09- to 56.45-fold, significantly greater than values for a single QTN, and RR_S_ values ranged from 1.39- to 19.38-fold, only slightly greater than values for a single QTN. The above results suggest that the disease resistant haplotype arising from a combo of two QTNs could significantly increase resistance performance without significantly increasing the disease risk of its carriers.

### QTN–QTN interaction and epistasis

According to the QTN–QTN interaction estimates calculated using Plink v1.07 (see **Supplementary Table S2**), among the 78 QTN–QTN pairs generated from the 13 QTNs shown in **Table 3** (1/2(13×12)), six pairs showed very significant epistatic effects (p<0.01) and nine pairs showed significant epistatic effects (0.01≤p<0.05). Among the eight combos (QTN–QTN pairs) listed in **Table 3**, three showed epistatic effects (0.01≤p<0.05), and the absolute SCR_R_ value was little greater than that of the more resistant QTN in the pair in each case, with differences of -0.1 (SCR_R_ of *S5_211.S8_123* minus *S8_123*), 0.3 (SCR_R_ of *S6_165.S6_164a* minus *S6_165*), and 0.5 (SCR_R_ of *S5_145.S5_210* minus *S5_145* or *S5_210*). Combo 2 (*S6_165.S8_123*), with SCRR=2.4, showed no significant epistatic effect (p = 0.1273). These findings indicate that epistasis or gene modification is quite common in the gene regulation network, but other types of interaction, such as additive effects, likely also play important roles in enhancing SCR resistance.

### Genomic and transcriptomic annotation of QTNs

**Figure 5:**
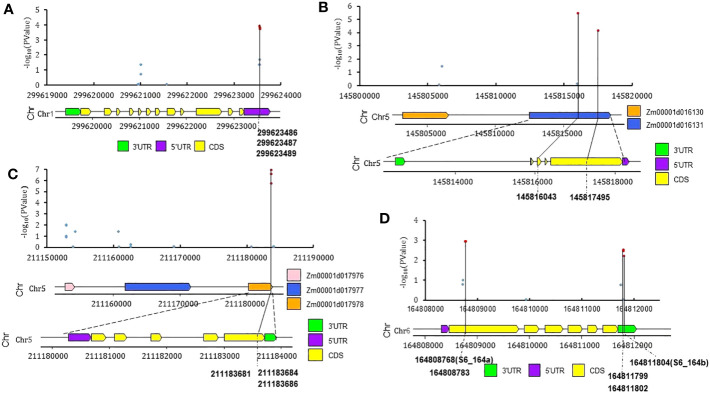
The polymorphic SNPs surrounding four significant QTNs and their SCR phenotype associations, distributions, and structure of candidate genes. **(A)** *Zm00001d034678* (*nbcs4*, near *S1_299b*); **(B)** *Zm00001d016131* (*GTE4*, near *S5_145*); **(C)** *Zm00001d017978* (*EG1*, near *S5_211*); **(D)** *Zm00001d038806* (*hsp101*, near *S6_164b*). Red dots represent -log_10_(PValue.QE) >2.

The associations between resistance to southern corn rust (SCR) and the polymorphic SNPs surrounding key significant QTNs were further examined using the intermediate results file via a scatter diagram. The relationships between associations and gene structures were studied using a gene structure diagram (**Figure 5**). The transcriptomic profiles of the candidate genes were obtained from maize transcriptomic databases via MaizeGDB (see Material and Methods section, **Supplementary Figures S3A-L**, **S4A-L**). **Supplementary Figure S2** shows the expression profiles of these candidate genes in different tissues, as obtained from MaizeGBD, and **Supplementary Figure S3** shows an organ-specific and stress-induced gene expression atlas for them, also retrieved from MaizeGDB. Here, we only list a select set of QTNs and putative candidate genes for further scrutiny.

(1) QTN *S1_218* (**Table 1**, V4: chr1:218619398, bin 1.07) was identified as significant by two GWAS models, with an LOD score of 4.58 and phenotype contribution of 8.1%. *S1_218* was located in the CDS region of *Zm00001d032244* (probable inactive receptor kinase) and A MYB-transcription factor 146 (*Zm00001d032240*) was located 110kb upstream; *Zm00001d032240* is notably upregulated after infection by *Cercospora zeina* (**Supplementary Figure S4A**) (Hoopes et al., 2019). MYB transcription factors have been reported to function as negative regulators of plant immunity (Wang et al., 2021) and positive regulators of the defensive response to stripe rust in wheat (Zhu et al., 2021), and have also been found to be associated with resistance to rust disease in foxtail millet (Bai et al., 2019).

(2) QTN *S1_299b* (**Figure 5A**, V4: chr1:299623487, bin 1.11): with an LOD score of 8.58 and phenotype contribution of 18.13%, this encodes a permease I (*Zm00001d034678*, *nbcs4*, nucleobase-cation symporter4). According to the results of genome scanning of the genome region of *Zm00001d034678* (*nbcs4*) (**Figure 5A**), three SNPs (Chr1: 299623551, 299623552, 299623554) with -log_10_ (Pvalue.Q)< 2 were located on the 5’ UTR region of *Zm00001d034678* (*nbcs4*); a high level of gene expression in leaves was also reported (**Supplementary Figure S3B**). One of the nucleobase-cation symporters (*NBCS*), named *MdNAT7*, has been reported to transport xanthine and uric acid, which enable more efficient scavenging of ROS by enhancing H_2_O_2_-scavenging enzymes in apple (Sun et al., 2021a).

(3) QTN *S2_12* (**Table 1**, V4: chr2:12916090, bin 2.01) was identified by two GWAS models, with an LOD score of 5.07 and phenotype contribution of 4.06%; an *rlk12* gene (Zm00001d002447, receptor-like protein kinase 12) was located 11kb upstream, and has been found to be a fungal resistance-related gene (Zhang et al., 2017b; Yang et al., 2019), with *Zm00001d002447* having been found to express in mature leaves and its level of expression to be elevated by fungal infection (**Supplementary Figure S3C**; **Supplementary Figure S4C**) (Hoopes et al., 2019).

(4) QTN *S5_145* (**Figure 5B**,V4: chr5:145816043, bin 5.04): with an LOD score of 3.47 and phenotype contribution of 11.16%, two SNPs (Chr5:145816043, 145817495) with -log_10_ (Pvalue.Q) > 2 were located on the CDS regions (the SNP chr5:145816043 is a synonymous SNV, and the SNP chr5:145817495 is a nonsynonymous SNV; **Supplementary Table S3**) of *Zm00001d016131* (Transcription factor *GTE4*); *Zm00001d016131* has been found to express in leaves, and its expression is elevated by fungal infection (**Supplementary Figure S3F**, **Figure S4F**) (Hoopes et al., 2019).

(5) QTN *S5_211* (**Figure 5C**, V4: chr5:211183684, bin 5.06) was identified by three GWAS models, with an LOD score 6.81 and phenotype contribution 11.69%. *S5_211* was located in the CDS region (synonymous SNV, **Supplementary Table S3**) of *Zm00001d017978* (*cellulase25*). An immediate-to-early fungal elicitor protein (CMPG1) encoding gene, *Zm00001d017976*, was located 280kb upstream; expression of *Zm00001d017976* was significantly elevated by *Colletotrichum graminicola* infection (**Supplementary Figure S4H**) (Hoopes et al., 2019).

(6) QTN *S6_164a* (V4: chr6:164808768, bin 6.07) and QTN *S6_164b* (V4: chr6:164811804, bin 6.07) were two QTNs found to be only 3036bp apart and tightly linked with high LD (**Supplementary Figure S1**). The two significant QTNs *S6_164a* (Chr6:164808768) and *S6_164b* (Chr6:164811804) were located in the CDS (synonymous SNV, **Supplementary Table S3**) and 3′UTR regions of *Zm00001d038806* (*hsp101*), respectively (**Figure 5D**); the expression of *Zm00001d038806* (*hsp101*) is notably upregulated when the temperature increases (**Figure S4K**) (Hoopes et al., 2019). Another gene named *rlk10* (*Zm00001d038791*, receptor-like protein kinase 10) was located 410kb upstream; the expression of *Zm00001d038791*(*rlk10*) has been found to be significantly elevated by *Colletotrichum graminicola* infection (**Supplementary Figure S4J**) (Hoopes et al., 2019). Overall, *Zm00001d038806* (*hsp101*) and *Zm00001d038791*(*rlk10*) are two potential candidate genes for these two QTNs.

(7) QTN *S8_123* (V4:chr8:123503579, bin 8.06) was identified by two GWAS models; two genes encoding Pgk2- phosphoglycerate kinase 2 (also known as *Metacaspase* type II, *Zm00001d010672*, *Zm00001d010673*) were located 300kb downstream and might be related to SCR resistance (Luan et al., 2020). *Zm00001d010672* has been found to be expressed in mature leaves and its expression is elevated by fungal infection (**Supplementary Figures S3K, L**; **Supplementary Figure S4L**) (Hoopes et al., 2019).

## Discussion

### Genetic mapping and genetic variant verification

Genetic mapping, either by linkage-based mapping or association-based mapping, mainly provides information on associations between DNA markers and trait phenotypic variation; further verification is required to establish that the association represents a causal relationship between underlying genetic variants and phenotypic variation. Traditional QTL mapping using linkage-based F_2_-derived populations is limited by a low frequency of chromosome recombination and low density of molecular markers; the QTLs identified are far away from the causal DNA variants, and intensive fine mapping is required to further pinpoint the precise chromosome location. A large number of SCR-related genetic loci have been reported since the 1960s, including some major effect QTLs on chromosome 10S: *RppM* (Wang et al., 2020a; Wang et al., 2022), *RppS/RppK* (Wu et al., 2015; Chen et al., 2022), *RppQ* (Chen et al., 2004), *RppC* (Deng et al., 2022), *RppP25* (Liu et al., 2003), *RppD* (Zhang et al., 2009), and *RppCML496* (Lv et al., 2020). Only a small number of QTLs, such as *RppC*, *RppK*, and *RppM*, have been verified by further genetic fine mapping and molecular studies (Chen et al., 2022; Deng et al., 2022; Wang et al., 2022). A set of genes/QTLs including *ZmREM1.3* (Wang et al., 2019b), *qSCR4.01* (Deng et al., 2020), *qSCR4.08* (Deng et al., 2019), *qSCR6.01*(Lu et al., 2020), *ZmMM1*(Wang et al., 2021), and *qSCR9.04* (Deng et al., 2019; Deng et al., 2020; Li et al., 2023b) have also been reported on chromosomes 2, 4, 6, 7, 8, and 9, but most of these have been identified based on preliminary linkage-based mapping data or comparative proteomic analysis. Association-based mapping strategies, such as GWAS, with a large mapping population and high-density SNP markers, have been shown to be very powerful in locating causal chromosome variation and precise DNA variants without further fine mapping, but are also more likely to generate a large number of false positive signals, and thus require extensive post-GWAS verification (Wen et al., 2018; Xiao et al., 2017; Wang et al., 2019a; Zhang et al., 2020; Li and Ritchie, 2021; Shikha et al., 2021). GWAS has been employed to identify SCR-related QTNs in several studies (Zhou et al., 2017; De Souza Camacho et al., 2019; Sun et al., 2022; Li et al., 2023b). The QTNs Chr4:173,863,109 and Chr6:169,030,253, identified by Sun et al. (2022), are approximately 4Mb away from the QTNs reported in this GWAS study: QTNs *S4_170* (Chr4:170324384) and *S6_164a* (Chr6:164808768), and QTNs *S6_164b*(Chr6:164811804) and *S6_165* (Chr6:165682422), respectively. *Zm00001d029980* (*mca1* - metacaspase 1, Chr1:97331766-97337093) and *Zm00001d047078* (*mca2* - metacaspase 2, Chr9:117546061-117551091) are genes related to disease resistance in the NLR (Rp1-D21)-mediated defense response (Luan et al., 2020). The NLR protein Rp1-D21 is coded by *Zm00001d023317* (*rp1*-resistance to Puccinia sorghi 1, Chr10:2861471-2865816). Zm00001d010672 and Zm00001d010673, as candidate genes of QTN *S8_123* in our study, encode phosphoglycerate kinase 2, which is also known as metacaspase.

Most of the QTNs identified on chromosomes 1, 2, 4, 5, 6, and 8 were found to be type B, type C, and type D QTNs with small effects. To minimize the risk of reporting false positive QTNs, six different multi-locus GWAS models from Zhang et al. (2020) were applied to generate a shortlist of significant QTNs; two association-based post-GWAS procedures (case–control sampling and allele/haplotype effect analysis) were then followed for verification. For ultimate confirmation that a statistical association represents a true causal relationship between the QTNs identified and the SCR-related phenotypes observed, additional molecular genetic tools, such as transgenic studies and genome-editing, or signal transduction cascade verification, are needed (Zhu et al., 2021; Chen et al., 2022; Deng et al., 2022; Wang et al., 2022).

### Genotyping and genotype data

One critical component for successful application of GWAS is genotyping. DNA markers with sufficient density and low LD are important for association mapping. Due to multiple occurrences of historical chromosomal recombination, the LD block in diversity panel of inbred lines is expected to be much shorter than in linage-based F_2_-derived populations; low LD together with high marker density render GWAS with a diversity panel an effective approach to genetic mapping. In the past, the trade-off between genotyping cost and marker density had always been an issue: although SSR and SNP-chip platforms are more cost-effective than complete sequencing methods such as NGS, they yield fewer markers and a lower marker density on chromosomes, and thus have lower mapping resolution. In this study, we solved this dilemma by using an NGS platform known as Genotyping by Sequencing (GBS), which uses the DNA methylation-sensitive enzyme ApeK1 to cut maize chromosomes. Because ApeK1 cuts more frequently at less methylated, gene-rich regions of the maize chromosomes, it generates more short DNA fragments and thus more DNA sequencing reads and more SNP calls. In this project, even though the GBS method only achieves 5X genome coverage on average, the real genome coverage can reach over 30X in gene-rich chromosomal regions and may yield over 700K SNP loci (Bradbury et al., 2007; Elshire et al., 2011; Glaubitz et al., 2014; Shu et al., 2021).

### Multi-locus GWAS models

The selection of appropriate statistical models to detect and measure association is critical to the success of GWAS. The models should be able to deal with various features of phenotypic and genotype data, such as continuity and normality of phenotypic data, population structure and kinship information in genotype data, and confounding from other covariables. The R software package provided by Zhang’s group, mrMLM.GUI V4.0.2 (Zhang et al., 2020), includes six multi-locus GWAS statistical models and a multiple-step algorithm for dealing with different data types and reducing false positives. Under the framework of multi-locus random-SNP-effect mixed linear modeling, each marker on the maize chromosome was first scanned for statistical significance and a less stringent Bonforroni correction was adopted in the statistical test. The significant marker loci identified were then incorporated into a new multi-locus genetic model; their effects were estimated by an empirical Bayes method, and all non-zero effects were further evaluated by the likelihood ratio test. Comparative studies have shown that this R package is more powerful than other methods both in terms of the detection of both strong and weak signals and in terms of lower output of false-positive signals (Wang et al., 2016; Tamba et al., 2017; Wen et al., 2018; Zhang et al., 2017a; Ren et al., 2018; Tamba and Zhang, 2018; Wang et al., 2019a; Zhang et al., 2020; Uffelmann et al., 2021; Park et al., 2022; Li et al., 2023a). As shown in **Table 1**; **Supplementary Table S1**, a large portion of QTNs were detected by more than one models, indicating that these QTNs have strong associations with the SCR trait.

### Post-GWAS analysis

As reported in other GWAS projects in human and maize, our GWAS also identified a large number of statistically significant SNP variants or QTNs (**Table 1**; **Supplementary Table S1**), and the majority of the QTNs were located in non-coding regions of a putative gene or maize genome regions of unknown biological function. Verification of QTNs identified by GWAS has always represented a major challenge, but great progress has recently been made, with various post-GWAS methods of analysis having been developed and some important genes for complex human diseases having been successfully identified (Li and Ritchie, 2021; Uffelmann et al., 2021). In this study, two association-based post-GWAS procedures were conducted: first, case–control sampling was performed to verify the QTNs identified by GWAS and to check the expected QTN genotype predicted by the phenotype for consistency; and second, the SNPs near the QTNs were collected, their haplotypes were generated, and haplotype–trait associations were examined (see **Figures 3**–**5**). Additionally, putative genes within a 300kb neighborhood of the QTN were taken as candidate genes, and their transcriptomic profiles and biological functions were examined. The genome-wide transcriptomic association study (TWAS) approach has gained in popularity over the years due to its distinct ability to connect SNP variants with expressed genes and complex trait phenotypes (Li and Ritchie, 2021; Uffelmann et al., 2021). However, the majority of QTNs identified in this study were located in non-coding regions of putative genes or non-genic regions of chromosomes; similar findings have also been reported in a number of studies of humans, rice, and maize, where the majority of trait-associated DNA markers are located in non-genic regions, and their genetic effects are generally greater than those in genic regions (Li et al., 2012; Rodgers-Melnick et al., 2016; Wei et al., 2020). Many studies have shown that complex cis- and trans-transcriptional regulatory mechanisms do not take effect within the coding regions of genes and more likely reside in the non-coding genomic neighborhood of identified QTNs (Li and Ritchie, 2021); thus additional approaches for post-GWAS analysis, such as transgenic validation, genome editing, and molecular function analysis, are necessary.

### Connection between QTNs and molecular breeding

In this study, we attempted to connect the QTNs identified by GWAS with molecular breeding practice. Firstly, the allele of each QTN or a haplotype of a QTN combo was assigned to one of the five categories HR, R, MR, S, or HS based on the value of its allelic (or haplotype) effect; subsequently, thirteen QTNs were grouped into four allelic effect types (A, B, C, and D) according to their combination of allele types. The two type A QTNs, *S5_145* and *S5_210*, both located on chromosome 5, were found to have large allelic effects; each contributed approximately 10% of SCR-related phenotypic variation (r^2%^), and their highly resistant (HR) alleles were present in all major SCR-resistant inbred lines in the diversity panel. The two type B, two type C, and 7 type D loci, located on chromosomes 1, 2, 4, 5, 6, and 8, were found to have small allelic effects, and likely provide a broad spectrum of SCR resistance to resistant inbred lines. Following this analysis, the phenotypic impact of each QTN (and its alleles) on its carrier was measured in terms of two statistics, relative resistance (RR_R_) and relative susceptibility (RR_S_; see Materials and Methods for definitions); these measure the resistance efficacy and disease risk conferred on the carrier by a resistant allele or a susceptible allele, enabling maize breeders to easily understand the benefits and risks of integrating a particular allele or QTN into their breeding materials or into new maize varieties. GWAS has been shown to be effective in identifying QTLs with small effects or conferring broad resistance (Xu et al., 2014; Zhou et al., 2017; Xu et al., 2018; De Souza Camacho et al., 2019; Zhang et al., 2019; Sun et al., 2022). Most published SCR-related QTL-mapping projects have used F_2_-derived segregated populations or their permanent versions, such as DHs and RILs from a genetic cross of a highly resistant (HR) parent × a highly susceptible (HS) parent; such studies have identified a number of QTLs associated with major effects on the short arm of chromosome 10, but very few small-effect QTLs have been reported and verified (Zhao et al., 2013; Lv et al., 2020; Wang et al., 2020a), indicating that linkage-based QTL-mapping using the HR × HS genetic cross method is less effective in identifying small-effect resistance genetic loci for broad resistance. Large-scale GWAS using a large, well-designed diversity panel of breeding lines and high-density SNP markers is likely to be a more effective way of discovering small-effect, broad SCR-resistance genetic loci.

## Data availability statement

The datasets presented in this study can be found in online repositories. The names of the repository/repositories and accession number(s) can be found below: European Variation Archive (EVA) at EMBL-EBI, accession number is PRJEB64281 (The European Bioinformatics Institute< EMBL-EBI).

## Author contributions

GS: Initiate the GWAS project, provide the guide to data analysis methodology, write the first draft and final draft of the manuscript. AifangW: Conduct multi-locus GWAS data analysis and transcriptomic annotation, preparation of all the Tables and Figures, take part in editing the manuscript. XW: Provide important inbred lines used in GWAS project, collect SCR phenotype data. JD give advice on data analysis and result interpretation, edit the manuscript. RC: Collect SCR phenotype data from the field experiment. FG and AifenW: provide inbred lines for GWAS and design and manage phenotype collection field experiment. TL: manage the SNP marker data collection project. YW: Initiate the GWAS project, provide the funding of the project, provide some important. germplasm for the diversity panel, provide the insight on application of GWAS result to molecular breeding of SCR resistance traits, edit the manuscript. All authors contributed to the article and approved the submitted version

## Supplementary material

Supplementary Figure 1 The LD value (D’) between significant QTNs.

Supplementary Figure 3 Tissue-specific expression profiles of candidate genes retrieved from maizeGDB.
