# Supplementary material for: Identification of southern corn rust resistance QTNs in Chinese summer maize germplasm via multi-locus GWAS and post-GWAS analysis
Source: Front Plant Sci. 2023 Sep 21;14:1221395. doi: 10.3389/fpls.2023.1221395 (PMC10552154; doi:10.3389/fpls.2023.1221395)
Supplement: Supplementary Figure 1 — The LD value (D’) between significant QTNs. [file DataSheet_1.zip › Supplementary materials/Figure S1.PDF]

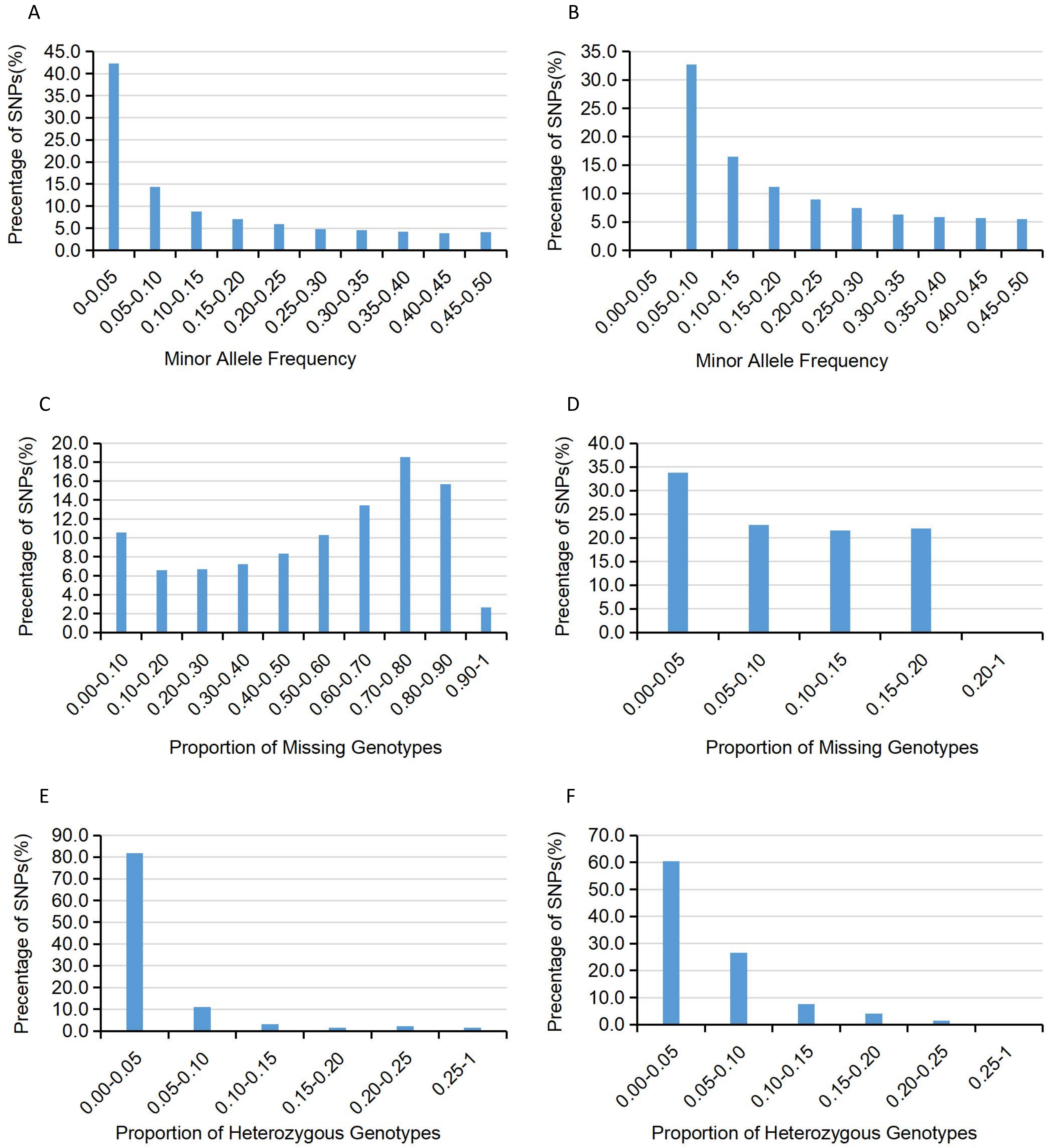

Figure S2. Distribution of minor allele frequency(MAF), proportion of missing and heterozygous genetypes in140 maize inbred lines based on unfiltered(876305) and filtered(73175) SNPs dataset. A. minor allele frequency of unfiltered SNP. B. minor allele frequency of filtered SNP. C. proportion of missing genotypes of unfiltered SNP. D. proportion of missing genotypes of filtered SNP. E. proportion of heterozygous genotypes of unfiltered SNP. F. proportion of heterozygous genotypes of filtered SNP.
