# Supplementary material for: Identification of southern corn rust resistance QTNs in Chinese summer maize germplasm via multi-locus GWAS and post-GWAS analysis
Source: Front Plant Sci. 2023 Sep 21;14:1221395. doi: 10.3389/fpls.2023.1221395 (PMC10552154; doi:10.3389/fpls.2023.1221395)
Supplement: Supplementary Figure 1 — The LD value (D’) between significant QTNs. [file DataSheet_1.zip › Supplementary materials/Figure S3.pdf]

A. S1_218: Zm00001d032240 (myb146)

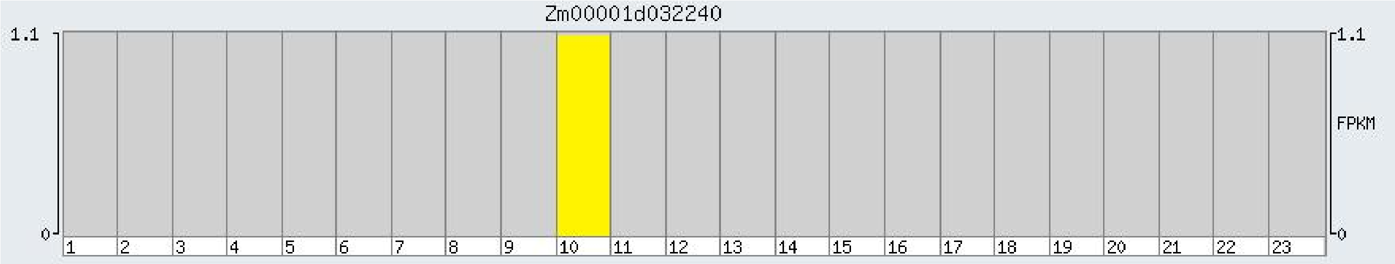

B. S1_299b: Zm00001d034678 (nbcs4)

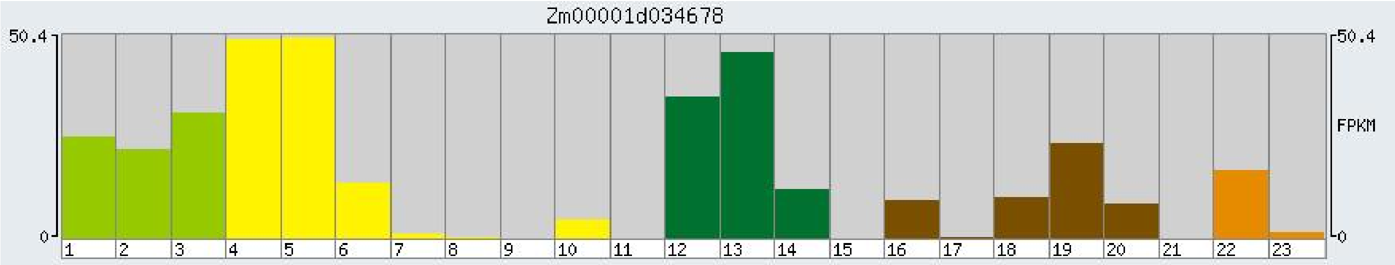

C. S2_12: Zm00001d002447 (WAK2)

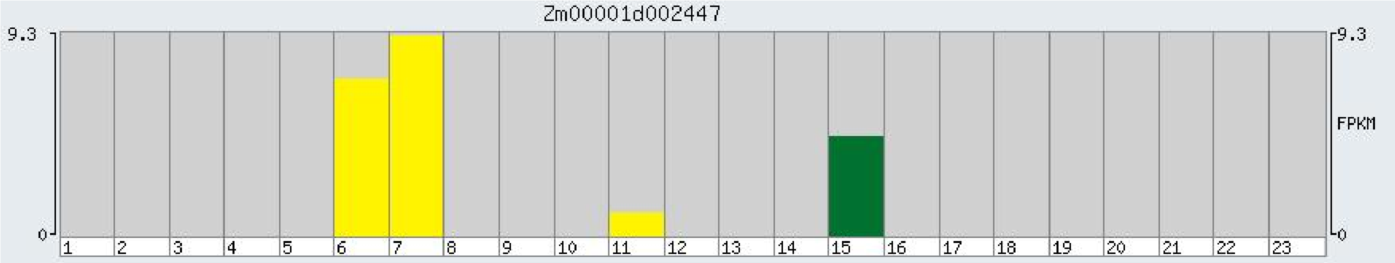

D. S4_170: Zm00001d051812 (hk6)

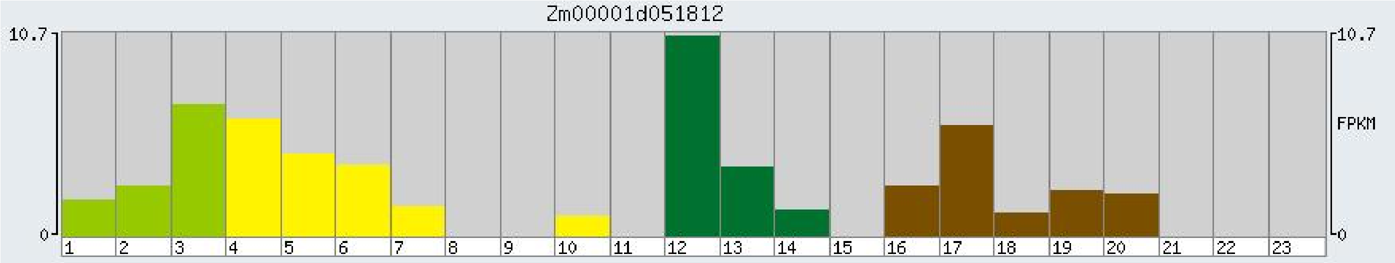

E. S4_200: Zm00001d052781 (cct23)

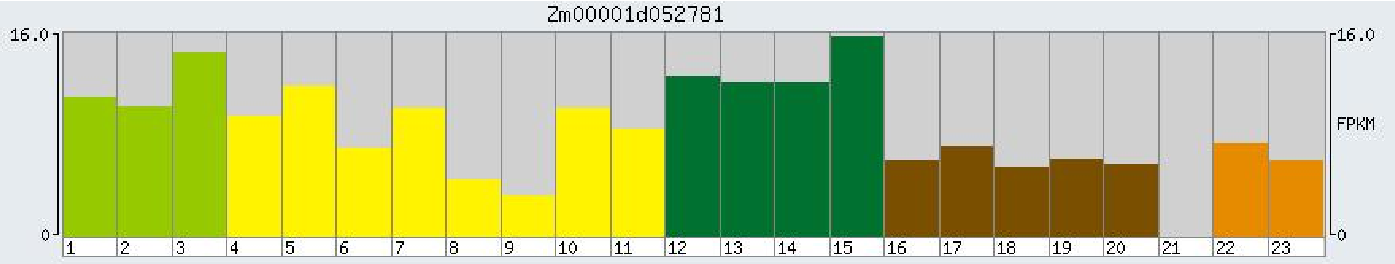

F. S5_145: Zm00001d016131 (GTE4)

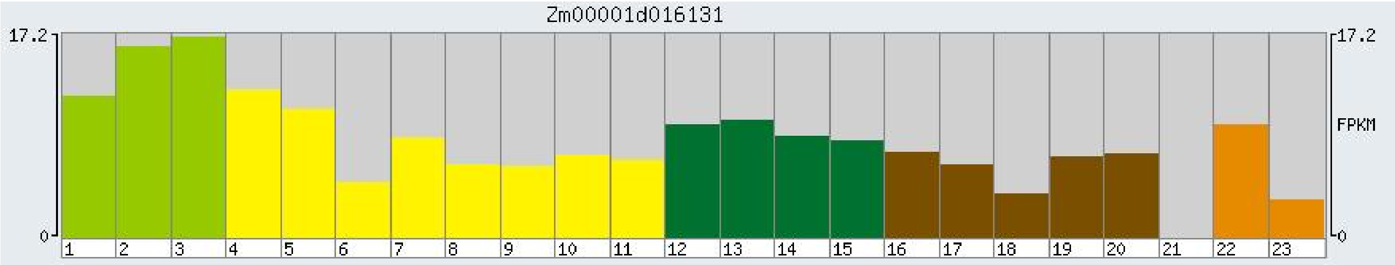

G. S5_210: Zm00001d017928 (almt3)

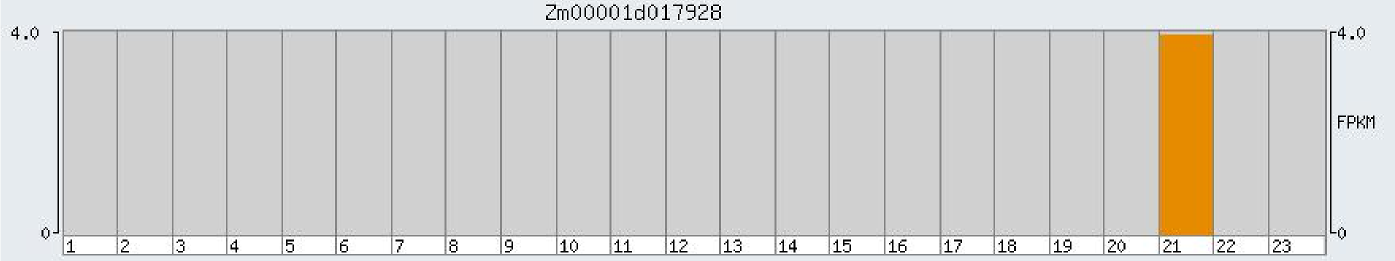

H. S5_211: Zm00001d017978 (EG1)

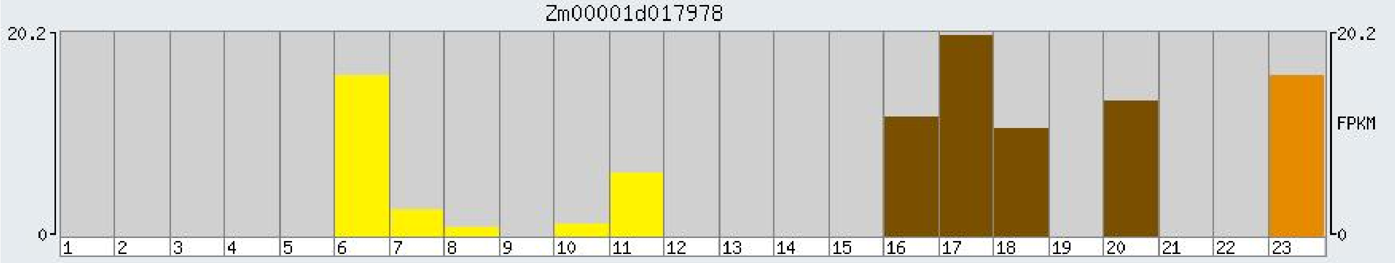

I. S6_164a+S6_164b: Zm00001d038806 (hsp101)

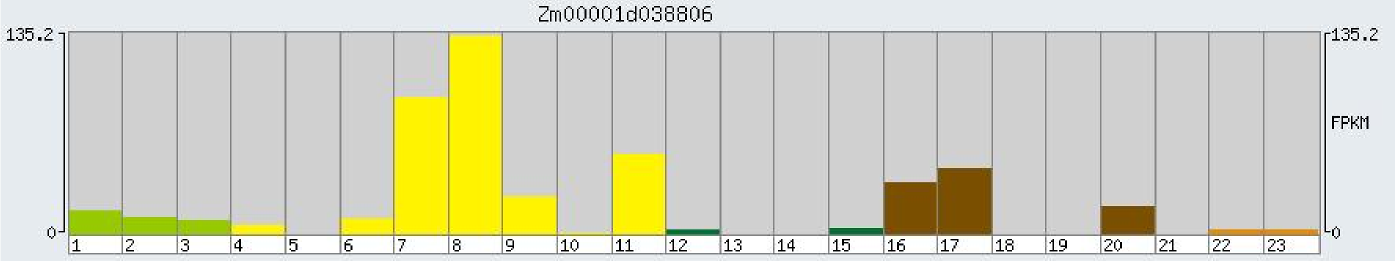

J. S8_123: Zm00001d010672 (pgk2)

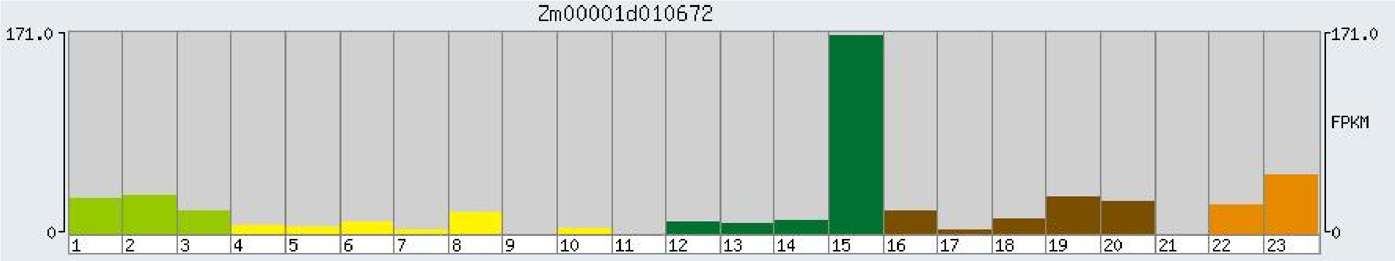

K. S8_123: Zm00001d010673 (pgk2)

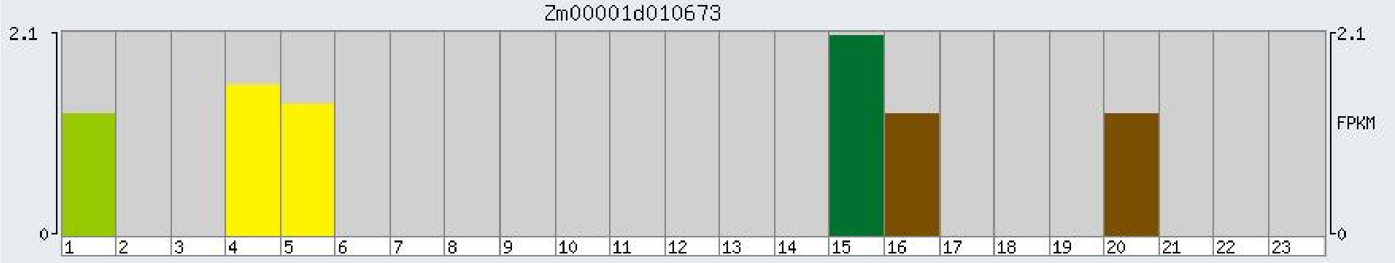

1. 6-7_Internode
2. 7-8_Internode
3. Meristem_16-19_Day
4. Ear_Primordium_2-4mm
5. Ear_Primordium_6-8mm
6. Embryo_20DAP
7. Embryo_38DAP
8. Endosperm_12DAP
9. Endosperm_Crown_27DAP
10. Germinatin_Kernels_2DAI
11. Pericarp/Aleurone_27DAP
12. Leaf_Zone_1_Symmetrical
13. Leaf_Zone_2_Stomatal
14. Leaf_Zone_3_Growth
15. Mature_Leaf_8
16. Primary_Root_5_Days
17. Root_Cortex_5_Days
18. Root_Elongation_Zone_5_Days
19. Root_Meristem_Zone_5_Days
20. Secondary_Root_7-8_Days
21. B73_Mature_Pollen
22. Female_Spikelet
23. Silk

Figure S4. Expression profiles of 11 candidate genes in different tissues retrieved from maize GDB
