# Supplementary material for: Identification of southern corn rust resistance QTNs in Chinese summer maize germplasm via multi-locus GWAS and post-GWAS analysis
Source: Front Plant Sci. 2023 Sep 21;14:1221395. doi: 10.3389/fpls.2023.1221395 (PMC10552154; doi:10.3389/fpls.2023.1221395)
Supplement: Supplementary Figure 1 — The LD value (D’) between significant QTNs. [file DataSheet_1.zip › Supplementary materials/Figure S4.PDF]

A. *S1_218*: *Zm00001d032240* (*myb146*)

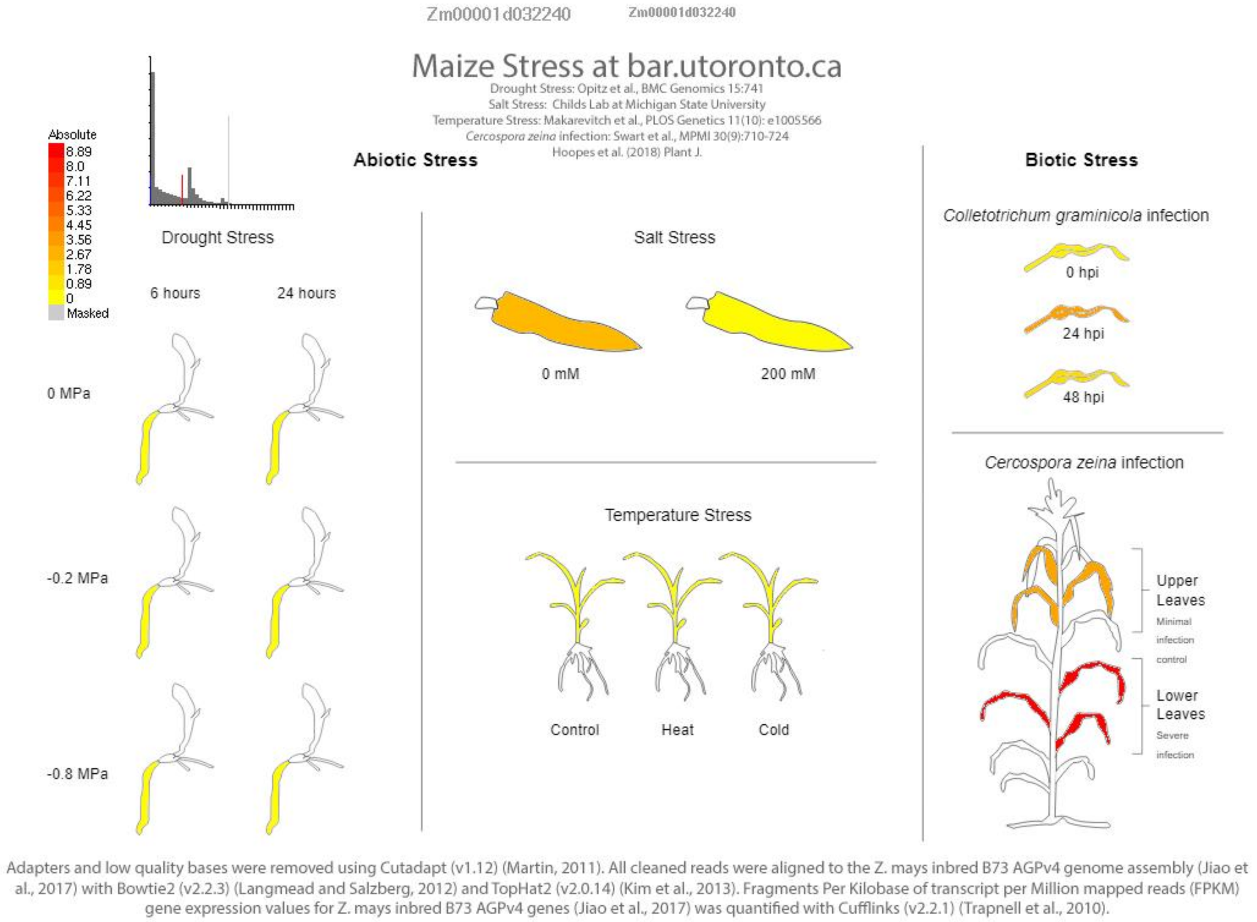

B. *S1_299b*: *Zm00001d034678* (*nbcs4*) No data

C. *S2_12*: *Zm00001d002447* (*rlk12*)

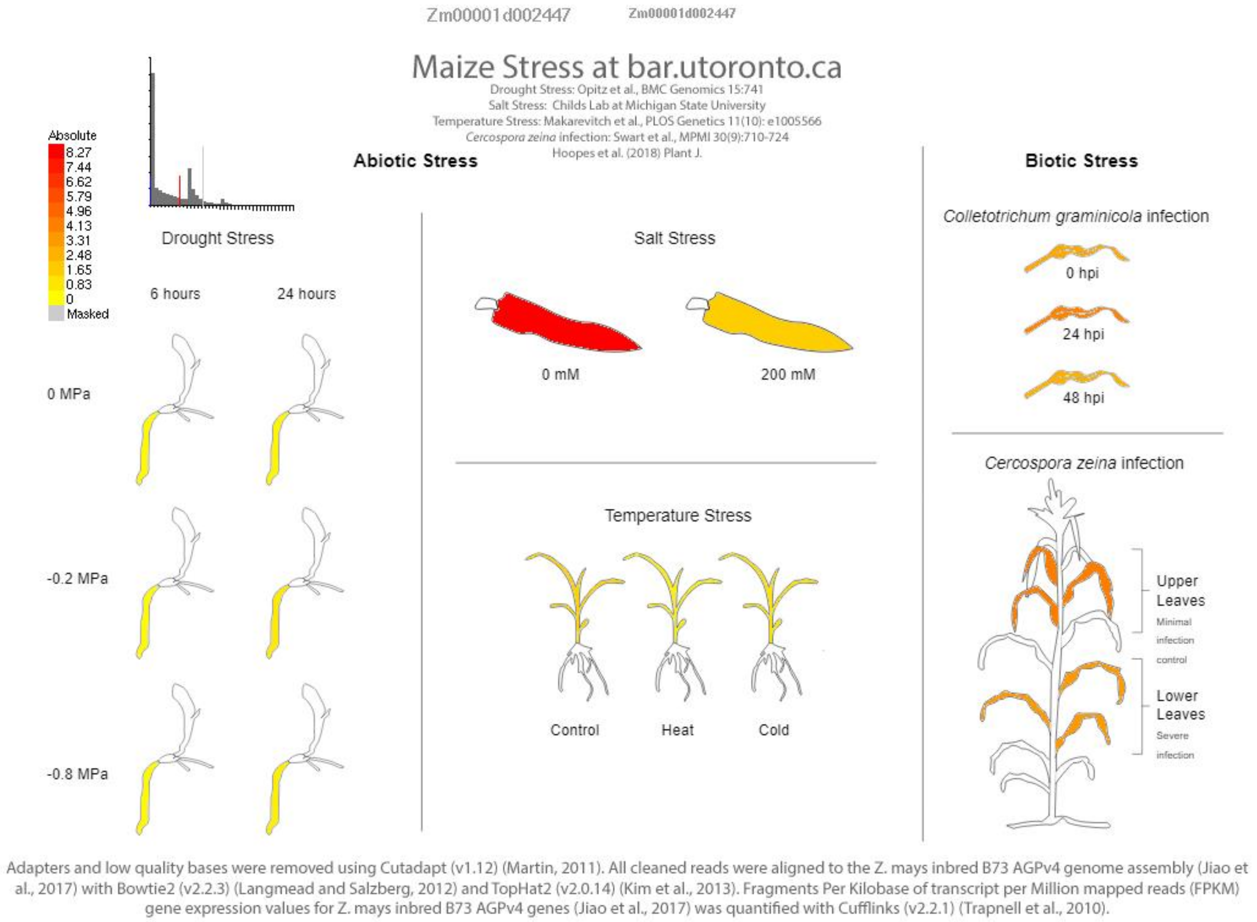

D. *S4_170*: *Zm00001d051812* (*hk6*)

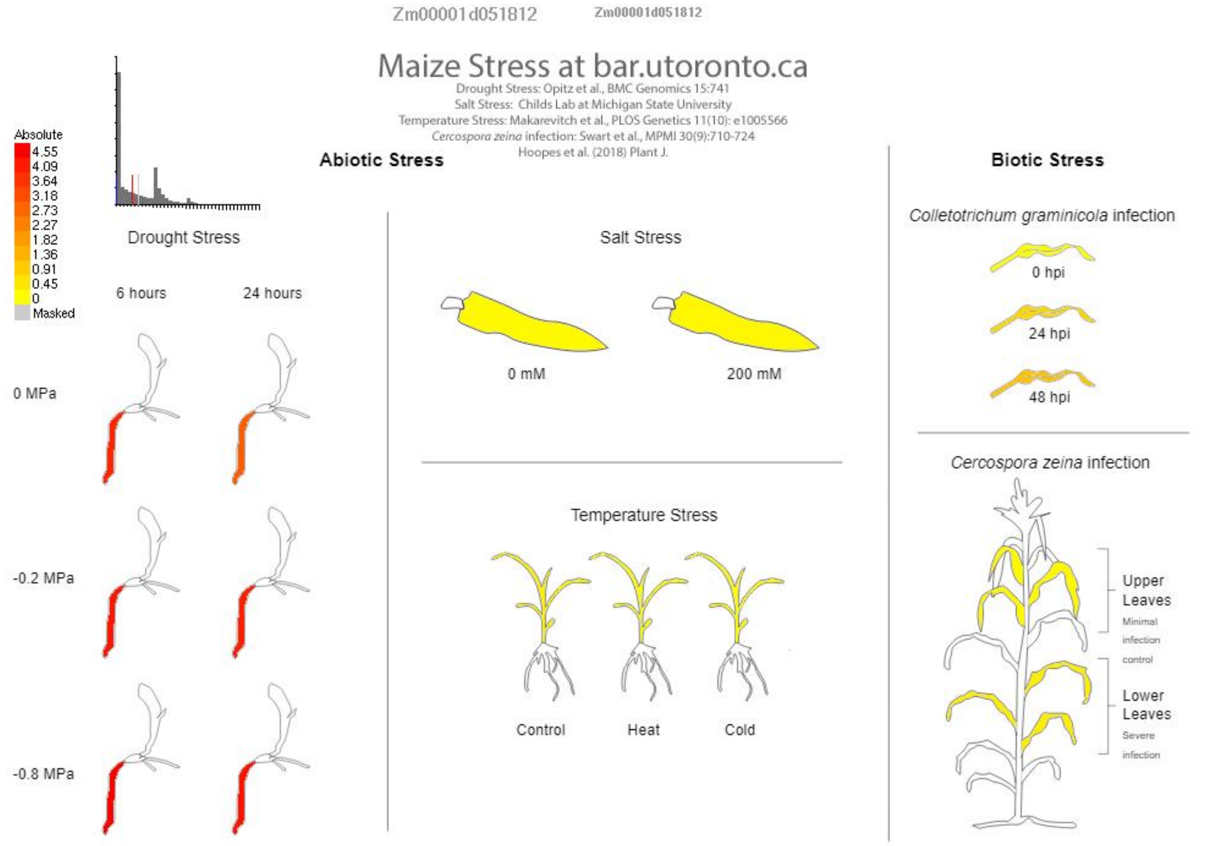

Adapters and low quality bases were removed using Cutadapt (v1.12) (Martin, 2011). All cleaned reads were aligned to the Z. mays inbred B73 AGPv4 genome assembly (Jiao et al., 2017) with Bowtie2 (v2.2.3) (Langmead and Salzberg, 2012) and TopHat2 (v2.0.14) (Kim et al., 2013). Fragments Per Kilobase of transcript per Million mapped reads (FPKM) gene expression values for Z. mays inbred B73 AGPv4 genes (Jiao et al., 2017) was quantified with Cufflinks (v2.2.1) (Trapnell et al., 2010).

E. *S4_200*: *Zm00001d052781* (*cct23*)

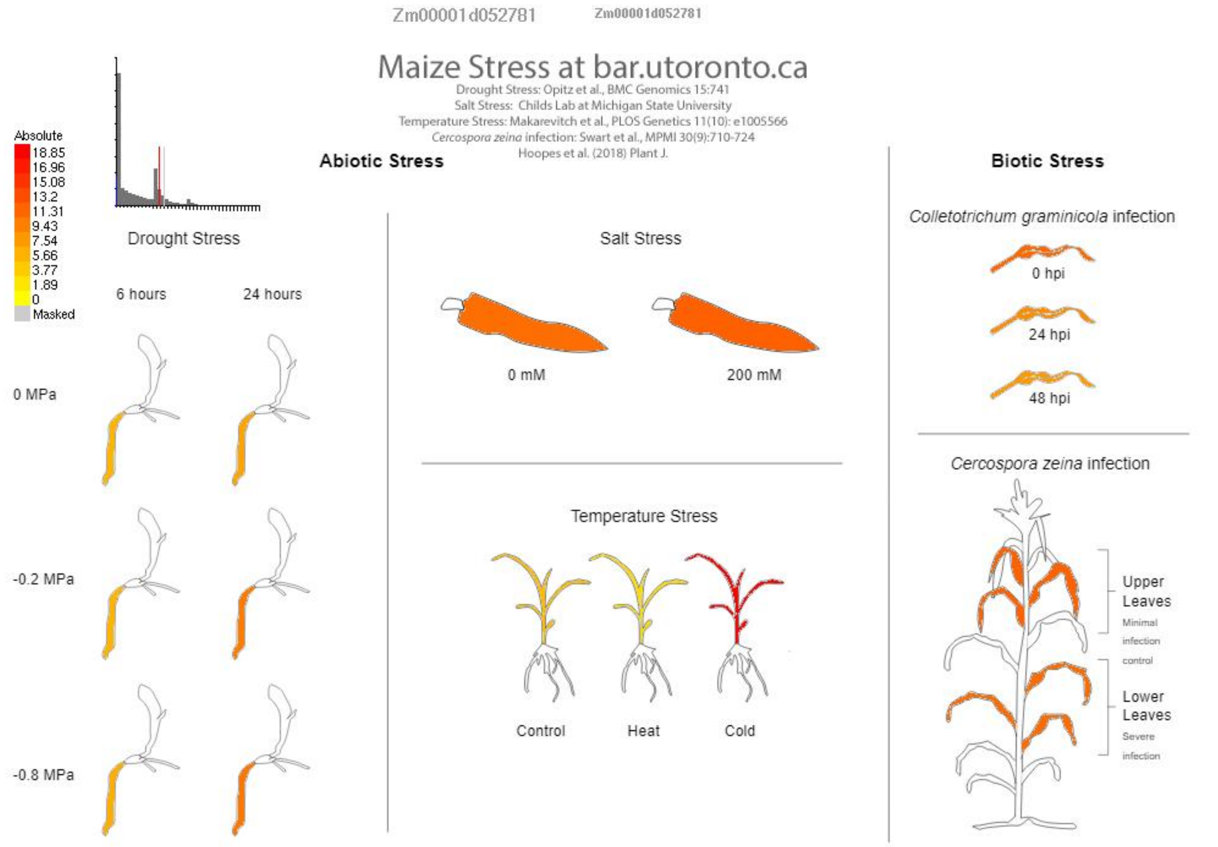

Adapters and low quality bases were removed using Cutadapt (v1.12) (Martin, 2011). All cleaned reads were aligned to the Z. mays inbred B73 AGPv4 genome assembly (Jiao et al., 2017) with Bowtie2 (v2.2.3) (Langmead and Salzberg, 2012) and TopHat2 (v2.0.14) (Kim et al., 2013). Fragments Per Kilobase of transcript per Million mapped reads (FPKM) gene expression values for Z. mays inbred B73 AGPv4 genes (Jiao et al., 2017) was quantified with Cufflinks (v2.2.1) (Trapnell et al., 2010).

F. *S5_145*: *Zm00001d016131* (*GTE4*)

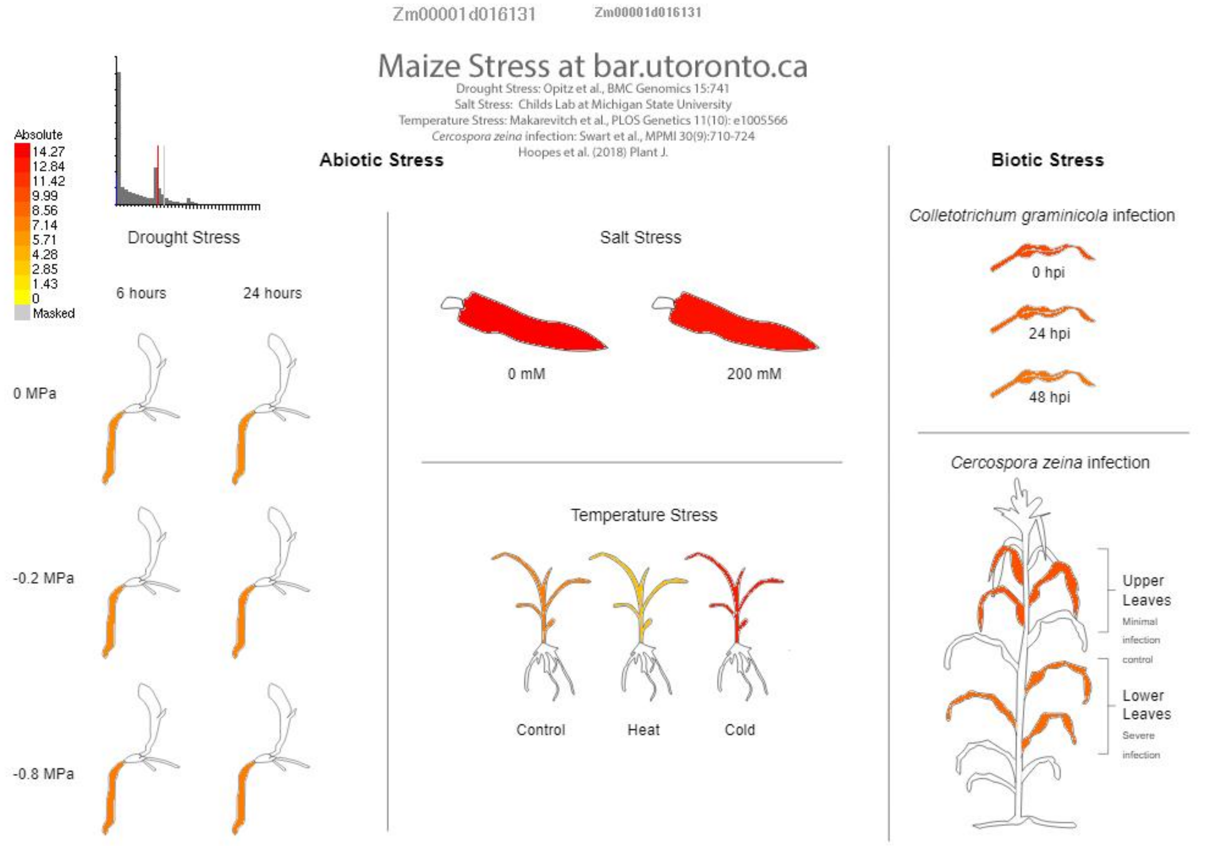

Adapters and low quality bases were removed using Cutadapt (v1.12) (Martin, 2011). All cleaned reads were aligned to the Z. mays inbred B73 AGPv4 genome assembly (Jiao et al., 2017) with Bowtie2 (v2.2.3) (Langmead and Salzberg, 2012) and TopHat2 (v2.0.14) (Kim et al., 2013). Fragments Per Kilobase of transcript per Million mapped reads (FPKM) gene expression values for Z. mays inbred B73 AGPv4 genes (Jiao et al., 2017) was quantified with Cufflinks (v2.2.1) (Trapnell et al., 2010).

G. *S5_210*: *Zm00001d017927* (*Fcf2*)

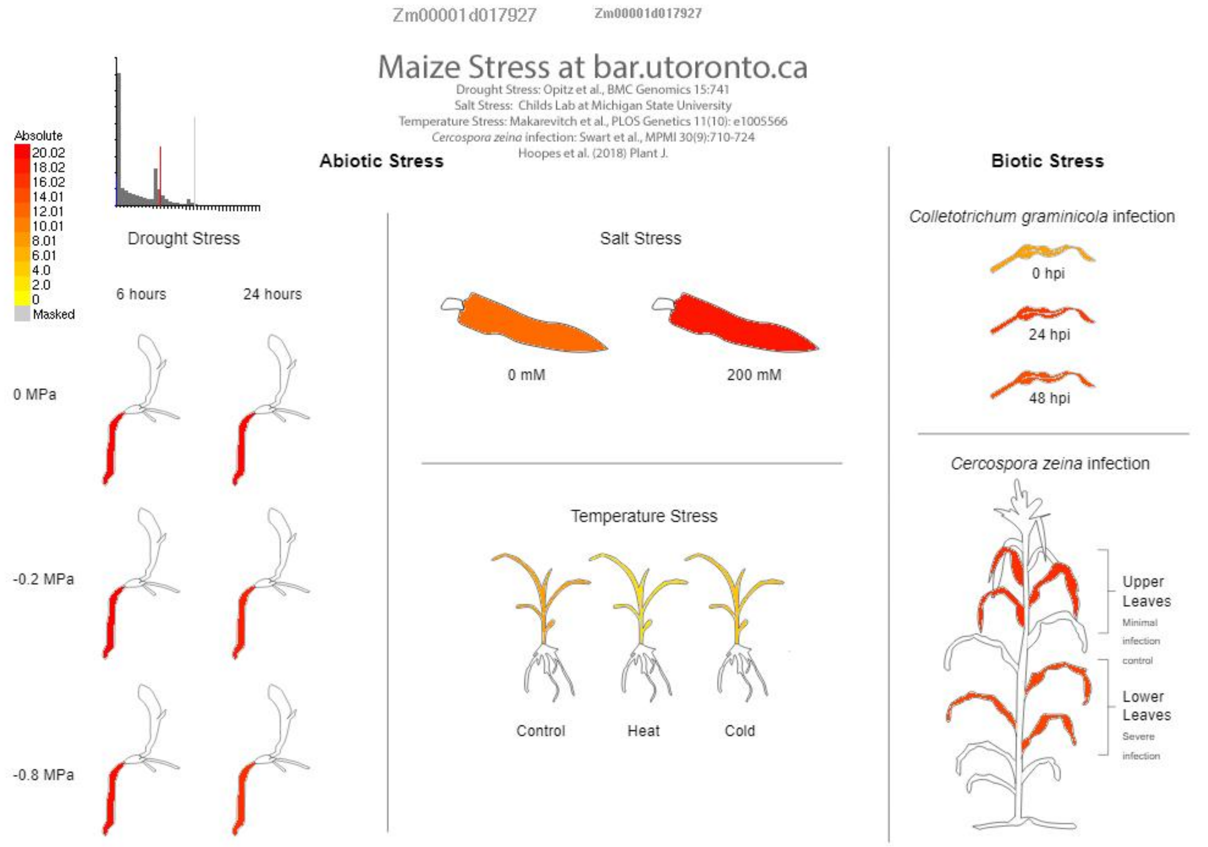

Adapters and low quality bases were removed using Cutadapt (v1.12) (Martin, 2011). All cleaned reads were aligned to the Z. mays inbred B73 AGPv4 genome assembly (Jiao et al., 2017) with Bowtie2 (v2.2.3) (Langmead and Salzberg, 2012) and TopHat2 (v2.0.14) (Kim et al., 2013). Fragments Per Kilobase of transcript per Million mapped reads (FPKM) gene expression values for Z. mays inbred B73 AGPv4 genes (Jiao et al., 2017) was quantified with Cufflinks (v2.2.1) (Trapnell et al., 2010).

H. *S5_211*: *Zm00001d017976* (*CMPG1*)

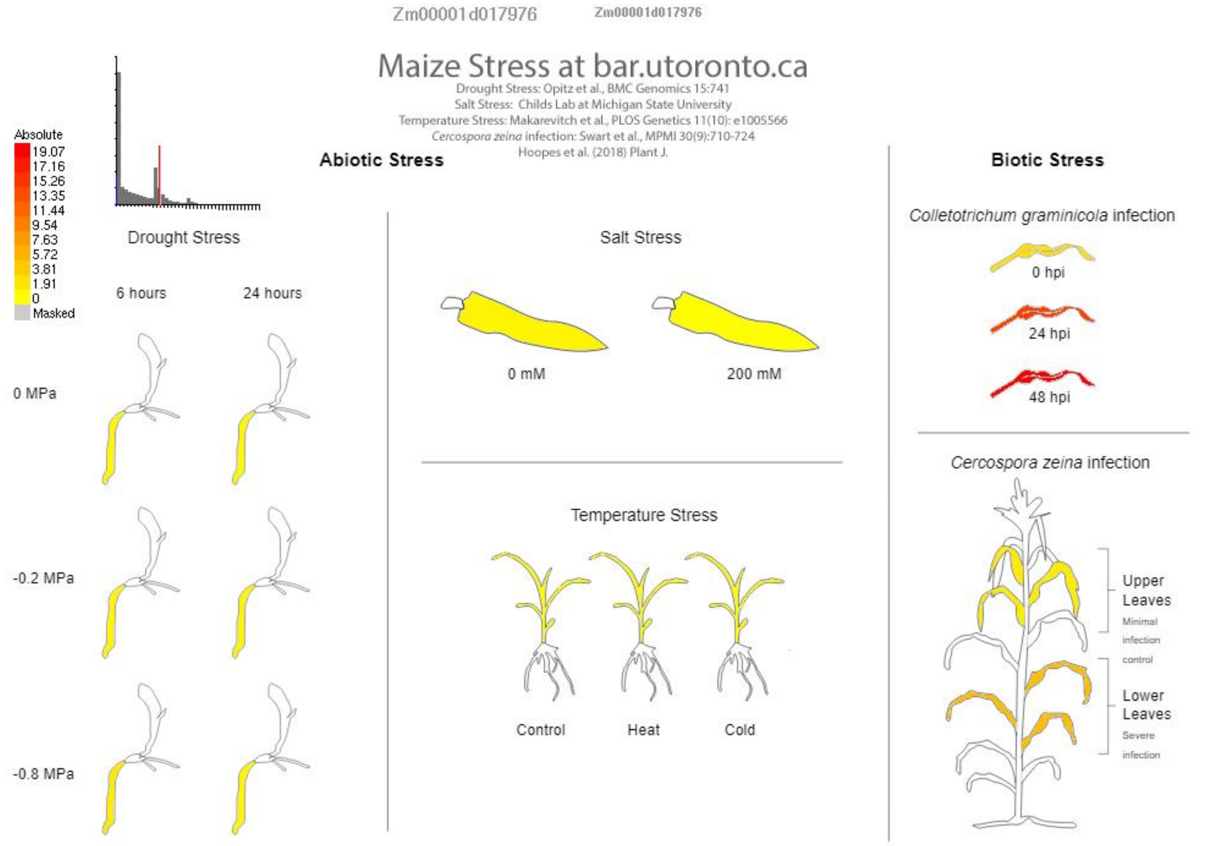

Adapters and low quality bases were removed using Cutadapt (v1.12) (Martin, 2011). All cleaned reads were aligned to the Z. mays inbred B73 AGPv4 genome assembly (Jiao et al., 2017) with Bowtie2 (v2.2.3) (Langmead and Salzberg, 2012) and TopHat2 (v2.0.14) (Kim et al., 2013). Fragments Per Kilobase of transcript per Million mapped reads (FPKM) gene expression values for Z. mays inbred B73 AGPv4 genes (Jiao et al., 2017) was quantified with Cufflinks (v2.2.1) (Trapnell et al., 2010).

I. *S5_211*: *Zm00001d017978* (*cel25*)

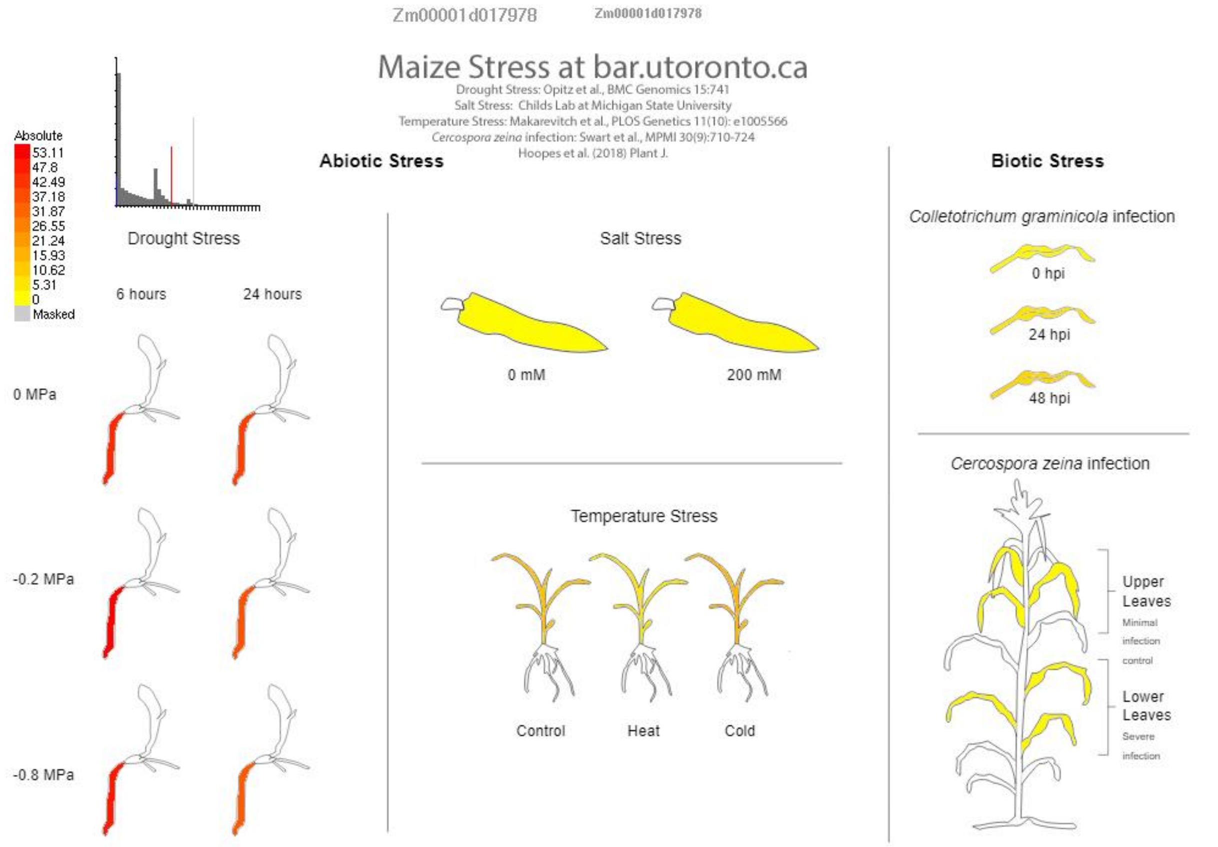

Adapters and low quality bases were removed using Cutadapt (v1.12) (Martin, 2011). All cleaned reads were aligned to the Z. mays inbred B73 AGPv4 genome assembly (Jiao et al., 2017) with Bowtie2 (v2.2.3) (Langmead and Salzberg, 2012) and TopHat2 (v2.0.14) (Kim et al., 2013). Fragments Per Kilobase of transcript per Million mapped reads (FPKM) gene expression values for Z. mays inbred B73 AGPv4 genes (Jiao et al., 2017) was quantified with Cufflinks (v2.2.1) (Trapnell et al., 2010).

J. *S6_164a+S6_164b*: *Zm00001d038791* (*rlk10*)

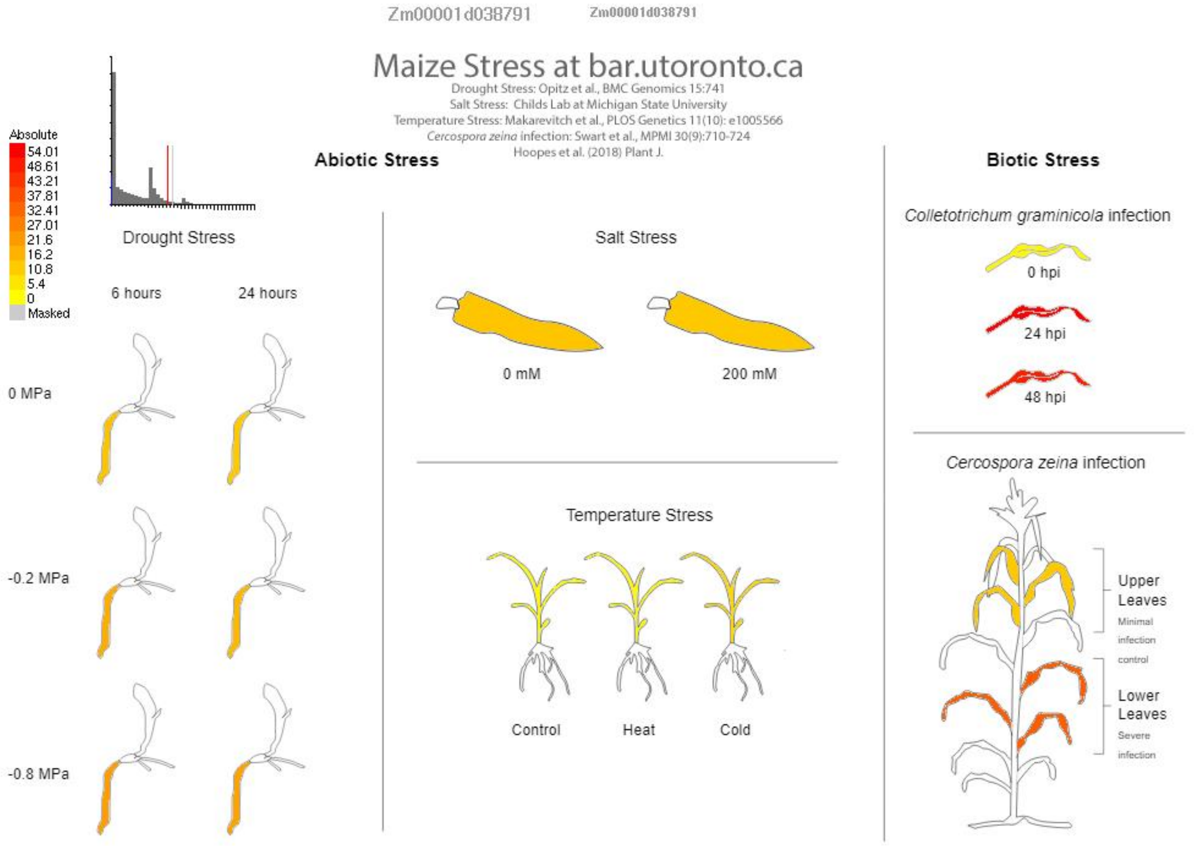

Adapters and low quality bases were removed using Cutadapt (v1.12) (Martin, 2011). All cleaned reads were aligned to the Z. mays inbred B73 AGPv4 genome assembly (Jiao et al., 2017) with Bowtie2 (v2.2.3) (Langmead and Salzberg, 2012) and TopHat2 (v2.0.14) (Kim et al., 2013). Fragments Per Kilobase of transcript per Million mapped reads (FPKM) gene expression values for Z. mays inbred B73 AGPv4 genes (Jiao et al., 2017) was quantified with Cufflinks (v2.2.1) (Trapnell et al., 2010).

K. *S6_164a+S6_164b*:*Zm00001d038806*(*hsp101*)

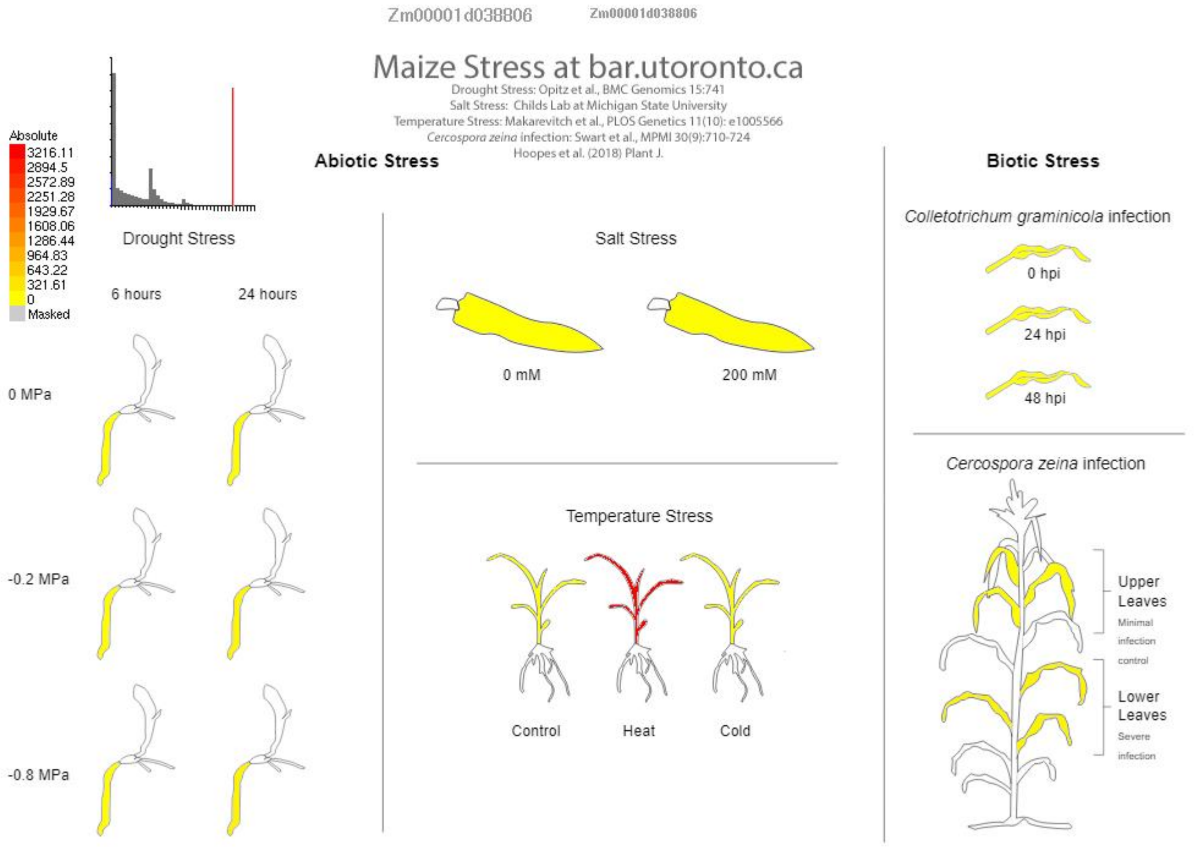

Adapters and low quality bases were removed using Cutadapt (v1.12) (Martin, 2011). All cleaned reads were aligned to the Z. mays inbred B73 AGPv4 genome assembly (Jiao et al., 2017) with Bowtie2 (v2.2.3) (Langmead and Salzberg, 2012) and TopHat2 (v2.0.14) (Kim et al., 2013). Fragments Per Kilobase of transcript per Million mapped reads (FPKM) gene expression values for Z. mays inbred B73 AGPv4 genes (Jiao et al., 2017) was quantified with Cufflinks (v2.2.1) (Trapnell et al., 2010).

L. *S8_123*: *Zm00001d010672* (*pgk2*)   (*Zm00001d010673* is as same as *Zm00001d010672*)

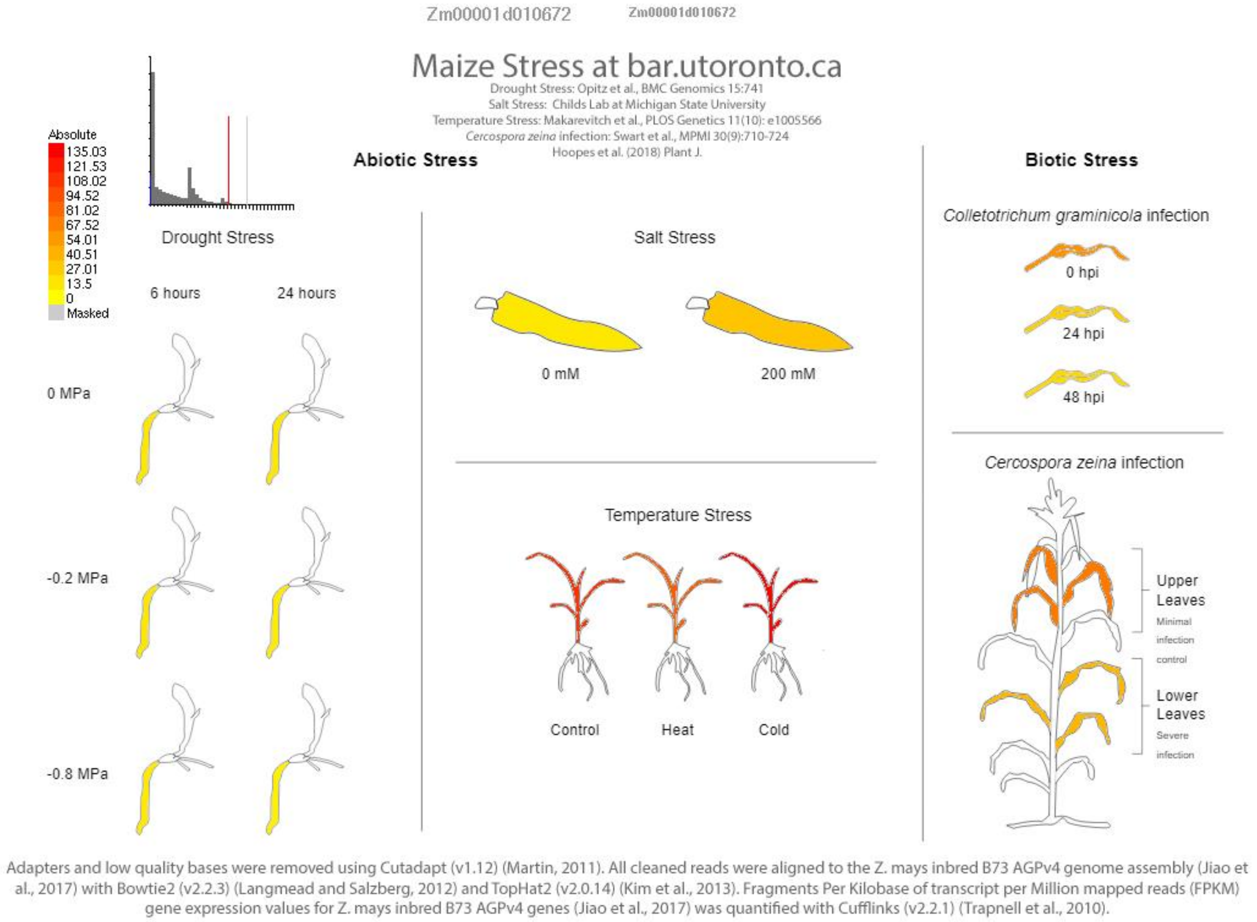

Figure S3. Maize organ-specific and stress-induced gene expression atlas of candidate genes from maizeGDB
