# Supplementary figures and images for: Identification of southern corn rust resistance QTNs in Chinese summer maize germplasm via multi-locus GWAS and post-GWAS analysis
Source: Front Plant Sci. 2023 Sep 21;14:1221395. doi: 10.3389/fpls.2023.1221395 (PMC10552154; doi:10.3389/fpls.2023.1221395)

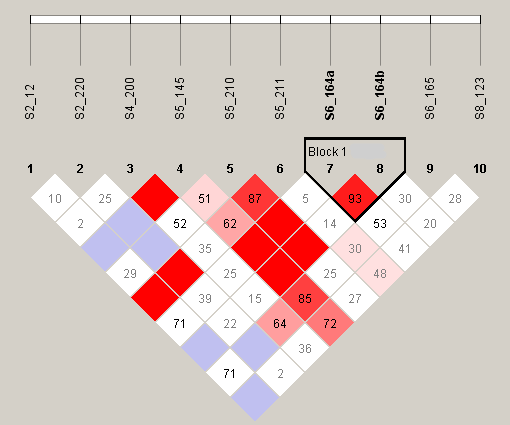

Supplement: Supplementary Figure 1 — The LD value (D’) between significant QTNs. [file DataSheet_1.zip › Supplementary materials/Figure S2.TIF]
